# AXL phosphosite mapping links FAK1 and YAP1 signaling to erlotinib resistance in EGFR-mutant lung cancer

**DOI:** 10.1016/j.celrep.2026.117717

**Published:** 2026-07-20

**Authors:** Marc Creixell, Scott D. Taylor, Jacqueline Gerritsen, Mingxuan Jiang, Song Yi Bae, Teresa Augustin, Michelle Loui, Carmen Boixo, Saki Minamino, Victoria Gong, Pau Creixell, Forest M. White, Aaron S. Meyer

**Affiliations:** 1 Department of Bioengineering, University of California, Los Angeles, Los Angeles, CA 90095, USA; 2 Jonsson Comprehensive Cancer Center, University of California, Los Angeles, Los Angeles, CA 90095, USA; 3 Department of Biological Engineering, Massachusetts Institute of Technology, Cambridge, MA 02139, USA; 4 Koch Institute for Integrative Cancer Research, Massachusetts Institute of Technology, Cambridge, MA 02139, USA; 5 Center for Precision Cancer Medicine, Massachusetts Institute of Technology, Cambridge, MA 02139, USA; 6 Cancer Research UK Cambridge Institute, University of Cambridge, Li Ka Shing Centre, Robinson Way, Cambridge CB2 2RE, UK; 7 Lead contact

## Abstract

Targeted therapies for receptor tyrosine kinases are effective but invariably limited by drug resistance. In EGFR-mutant lung cancer, AXL activation drives resistance to erlotinib by restoring cell survival and migration. To map these signaling mechanisms, we generated a panel of lung adenocarcinoma PC9 cell lines with phenylalanine substitutions at each intracellular AXL tyrosine residue. By integrating phosphorylation data with phenotypic changes via multivariate modeling, we found that AXL signaling organizes into two clusters enriched for Abl1 and SFK substrate motifs. A peptide specificity screen identified FAK1 as a top proximal substrate of AXL. Downstream, AXL-mediated YAP1 activation was found to sustain drug tolerance, while combined inhibition eliminated persister cells *in vitro*. These AXL and YAP pathways correlate with disease progression and poor clinical outcomes in erlotinib-treated patients. Collectively, this study dissects the specific signaling regulators by which AXL drives erlotinib resistance.

## In brief

Creixell et al. map the early signaling networks triggered by AXL during EGFR inhibition in lung cancer cells. They reveal that AXL activates FAK1, SFK/Abl1, and downstream YAP to promote cell survival and migration. Co-inhibition of these pathways eliminates drug-tolerant persister cells.

## Graphical Abstract

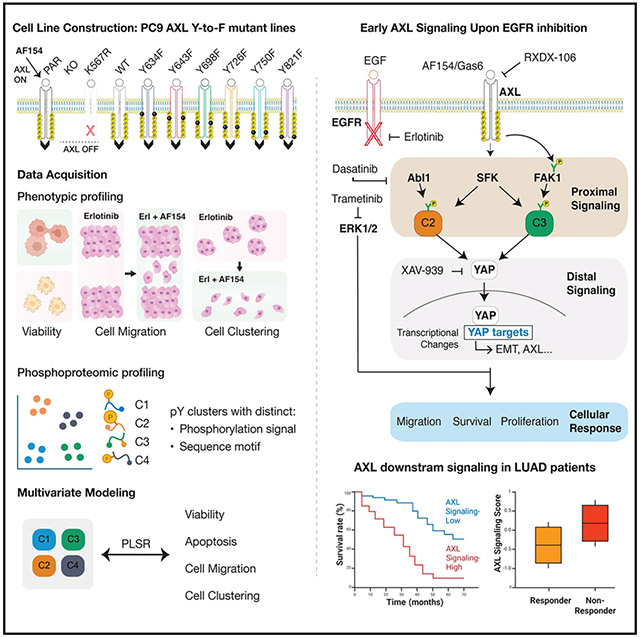

## INTRODUCTION

Lung cancer is the leading cause of cancer mortality, accounting for nearly 25% of all cancer deaths in the United States in 2022.^1^ Comprehensive genomic sequencing and expression profiling of non-small cell lung cancer (NSCLC) patients has revealed oncogenic driver mutations that can be therapeutically targeted, improving efficacy and tolerability relative to conventional chemotherapy. One such therapy is erlotinib, an EGFR tyrosine kinase inhibitor (TKI), which is effective in patients with advanced EGFR-mutant (EGFRm) NSCLC.^2^ However, responses to EGFR-targeted therapies are rarely curative, and most patients ultimately relapse due to acquired resistance.

AXL, a member of the Tyro3, AXL, and MerTK (TAM) receptor tyrosine kinase (RTK) family, is frequently upregulated in tumors that have acquired resistance to chemotherapy, targeted therapies, or immunotherapy, including EGFR-driven NSCLC.^3-13^ In EGFRm lung cancer, AXL expression is linked to epithelial-to-mesenchymal transition (EMT), enhanced migration, metastatic spread, and the survival of osimertinib- and erlotinib-resistant cells, including drug-tolerant persistent populations.^14-22^ Combination strategies targeting EGFR and AXL reverse resistance in preclinical models, positioning AXL as a promising therapeutic target.^7,21,22^ AXL-targeting agents including selective TKIs (bemcentinib, dubermatinib), ADCs (BA3011, ADCT-601, NCT05389462), and ligand traps (batiraxcept) have shown manageable safety and early antitumor activity, especially in biomarker-enriched populations and in combination regimens.^23-29^ Yet none of these drugs have been approved, and several first-generation programs have been discontinued (DS-1205, enapotamab vedotin).^23-29^ Despite substantial evidence implicating AXL in drug resistance and disease progression, it remains unclear, which specific downstream pathways AXL engages and how AXL-mediated resistance arises. Thus, a more comprehensive characterization of AXL downstream signaling could help refine biomarker-based strategies for patient selection.

Dissecting these pathways is complicated due to RTK cross talk and signaling pleiotropy: each RTK regulates multiple downstream pathways, many of which can also be activated by other RTKs. Approaches that directly perturb phosphorylation sites can help resolve this pleiotropy into pathway-specific effects. Because phenylalanine (F) is a non-phosphorylatable mimetic of tyrosine (Y), Y-to-F mutational studies can probe the signaling roles of individual phosphosites.^30^ For instance, AXL drives resistance to cetuximab and radiation through interaction of Abl1 and AXL Y821 in head and neck cancer and editing Y821 to F abolishes this resistance.^31^

Here, we combine systematic mutagenesis, phosphoproteomics, and computational modeling to define early AXL downstream signaling in EGFR-targeted lung cancer cells before overt drug resistance emerges. We generated a panel of PC9 derivatives, each harboring a single AXL Y-to-F mutation, and quantified phosphorylation, viability, apoptosis, migration, and an erlotinib-induced cell clustering phenotype in AXL-activated cells. Integrative modeling revealed that AXL activates Src-family kinases (SFKs), Abl1, and FAK1 as upstream components of the YAP pathway to promote cancer cell fitness. An AXL signaling signature correlated with AXL protein expression and YAP activation in EGFRm tumors, and both AXL and YAP signatures were enriched in erlotinib-treated EGFRm LUAD patients with metastatic, progressive disease. These analyses extend our findings *in vitro* and support the clinical relevance of AXL-YAP signaling in AXL-high EGFRm lung cancer patients.

## RESULTS

### AXL’s capacity to induce proliferation and migration varies across AXL Y-to-F mutants

**Figure 1.**
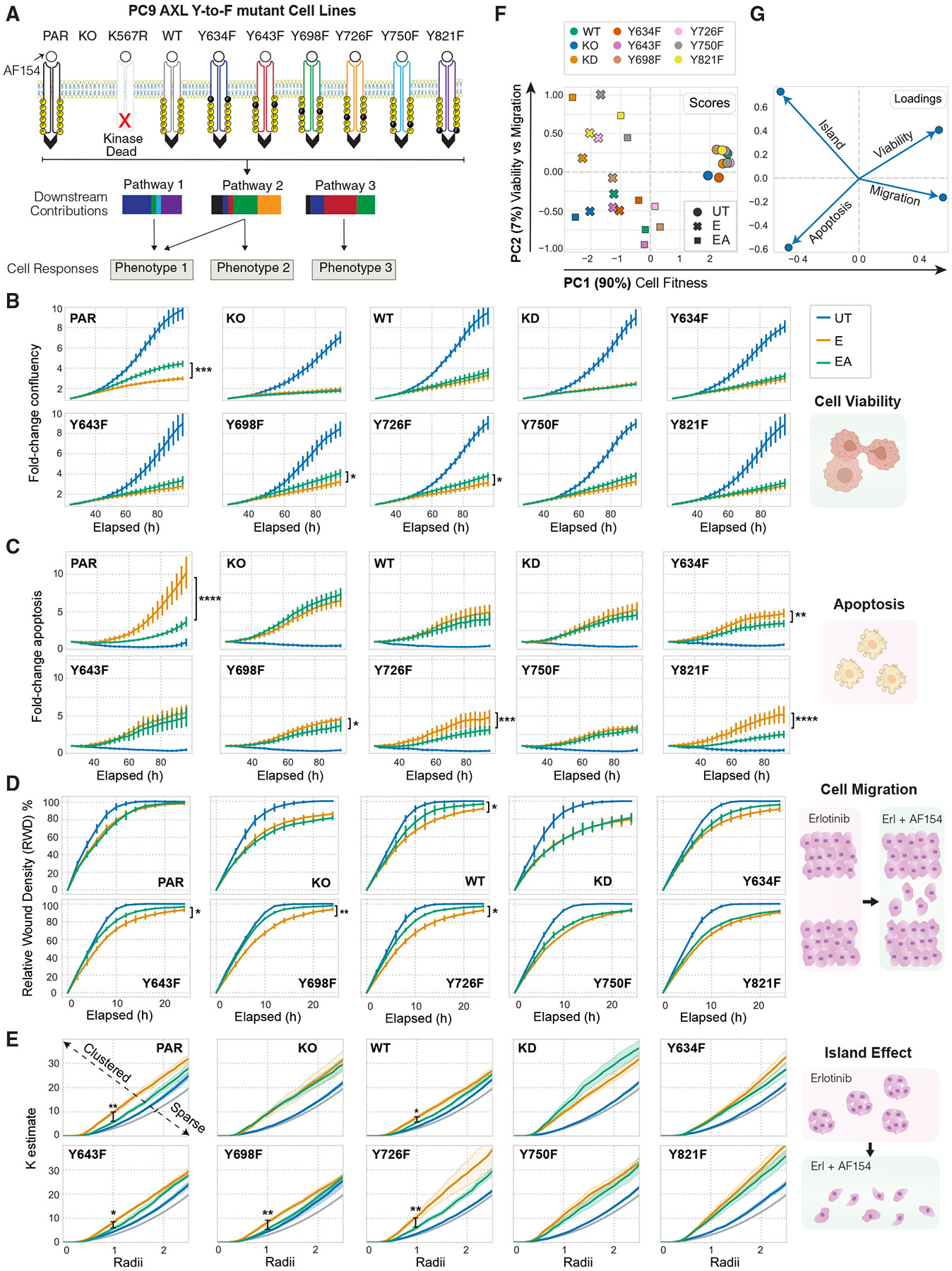
Oncogenic phenotypes vary across PC9 AXL Y-to-F mutants (A) Schematic of AXL Y-to-F mutant cell lines and treatment conditions with erlotinib (E) or erlotinib plus AXL-activating antibody AF154 (EA). (B–C) Live-cell imaging quantification of proliferation and cell death over 96 h. (D) Relative wound density from scratch-wound migration assays. (E) E-induced cell island effect quantified by Ripley’s K function at a radius of 1 μm, followed by a t-test comparing treatment conditions. (F–G) PCA scores and loadings summarizing relationships among proliferation, apoptosis, migration, and cell clustering. To assess statistical significance, E- and EA-treated conditions were compared across measured cell viability, death, and migration time points using t-tests (A–D). For all phenotypic measurements *n* = 3. * *p* < 0.05, ** *p* < 0.01, *** *p* < 0.001, **** *p* < 0.0001; ns, not significant.

To isolate AXL pathways driving specific phenotypes, we generated a panel of stable AXL Y-to-F mutant PC9 cell lines and measured phosphoproteomic and phenotypic responses during EGFR inhibition and AXL activation. As outlined in Figure 1A, different Y-to-F mutations were used as perturbations expected to differentially impact downstream pathways and phenotypes. By reconstructing AXL-specific variance with peptide clustering and partial least-squares regression (PLSR), we associated signaling changes with phenotypic outputs. Our goal was not to assign effects to individual tyrosines, but to systematically perturb AXL signaling and use multivariate modeling to holistically link AXL-driven signaling states to specific phenotypes.

A pilot selection-based fluorescence-activated cell sorting (FACS) assay in HCC827 along with existing knowledge of these sites in the literature,^4,31^ led us to choose positions 634, 643, 698, 726, 750, and 821 to generate stable AXL Y-to-F mutant cell lines in PC9 (Figures S1A and S1B). PC9 cells enabled controlled AXL activation during EGFR inhibition because they express minimally active baseline AXL and are erlotinib-sensitive.^20^ We knocked out AXL in PC9 parental (PAR) cells using CRISPR-Cas9 and lentivirally transduced either AXL wild-type (WT), kinase dead (KD), or a Y-to-F AXL mutant insert (Figure 1A). PC9 transgenic AXL cell expression and downstream signaling was confirmed using flow cytometry and biochemical assays (Figures S1C and D) (see supplemental information and limitations of the study).

We measured the ability of the different AXL mutant cell lines to proliferate, survive, migrate, and form cell “islands” in the presence of erlotinib (E, 1 μM) or the combination of E and the AXL-activating antibody AF154 (A, 300 ng/mL) (EA) (Figure 1A). As shown in our prior work,^20,32,33^ we used AF154 to transiently and maximally activate AXL, enabling us to capture the subsequent signaling response (see supplemental information).

We monitored cell proliferation and cell death using live cell imaging over 96 h of treatment with either E or EA (Figures 1B and 1C). We observed a significant increase in cell proliferation and decrease in apoptosis in EA-treated PC9 PAR cells compared to the E condition. WT cells followed the same trend with lesser magnitude, likely because each PC9 AXL cell line still had a fraction of AXL KO cells (Figure S1C). As expected, EA did not enhance proliferation or survival versus E in AXL KO and KD. While the mutants Y643F and Y750F effectively behaved like AXL KO or KD, Y698F, and Y726F cells promoted proliferation and prevented apoptosis upon AXL activation. On the contrary, when EA-treated, Y821F and Y634F cells failed to increase cell proliferation and yet significantly blocked apoptosis (Figures 1B and 1C).

We performed a scratch-wound assay to evaluate the migratory capacity of each cell line after treatment. PC9 PAR cells treated with E unexpectedly migrated similarly with or without the AXL-activating antibody, while KO and KD cells showed blocked migration regardless of treatment. Mutations Y643F, Y698F, and Y726F increased motility upon receptor activation, whereas Y634F, Y750F, and Y821F did not (Figure 1D). The induction of migration by E can be at least partially explained by the fact that E activates AXL even in the absence of A (Figure S1F).

Evaluating other phenotypes, we observed that at 48 h, E induced a sustained cell clustering effect. Cells established cell-to-cell adhesions and formed small “cell islands” (Figure 1E; Figure S1G). To quantify cell clustering, we used Ripley’s K function, a spatial clustering metric commonly used in astronomy to model whether objects like stars are found closer together than expected by chance.^34,35^ This algorithm enabled us to test whether PC9 cells distributed spatially randomly, against the null hypothesis of random distribution. Consistent with our initial observation, we found that PC9 PAR cells clustered more than expected within a 1 μm radius in response to E; however, AXL activation partially reversed this clustering. Y-to-F mutant cell lines showed a trend opposite to that seen in the scratch wound assay: Y643F, Y698F, and Y726F behaved like PC9 PAR and WT cells, while Y634F, Y750F, and Y821F remained clustered after EA treatment (Figure 1E).

We used principal-component analysis (PCA) to examine relationships among the four phenotypes (Figures 1F and 1G). PC1, representing most of the variation, separated untreated cells from both E and EA and partially separated EA from E cells. We used this component to represent overall cell fitness, which increases along PC1 for all mutants treated with A except for KO and KD, where A had no effect (Figure 1F, scores). Phenotypes increased by AXL activation are positively associated with PC1, while those decreased are negatively associated. PC2 separated AXL-induced viability from migration, with higher PC2 scores reflecting increased viability/decreased apoptosis but reduced migration, and the converse in the opposite direction (Figure 1G, loadings). Accordingly, WT, Y643F, Y698F, Y726F, and Y750F shifted positively along PC1 and negatively along PC2 (migration-driven effects), whereas Y634F and Y821F shifted positively along both components (anti-apoptotic effects).

Overall, these results suggest each Y-to-F mutant affects cell viability, death, migration, and clustering differently. Some mutants, like Y750F, blocked all phenotypic effects, behaving like KO and KD cells. Y698F and Y726F cells behaved like PC9 PAR and WT cells, with little impact on outcomes. Others, like Y634F and Y821F, showed the most varied effects, blocking AXL-induced viability, migration, and scattering while enhancing cell survival.

### AXL mutants selectively disrupt downstream pathway effects

**Figure 2.**
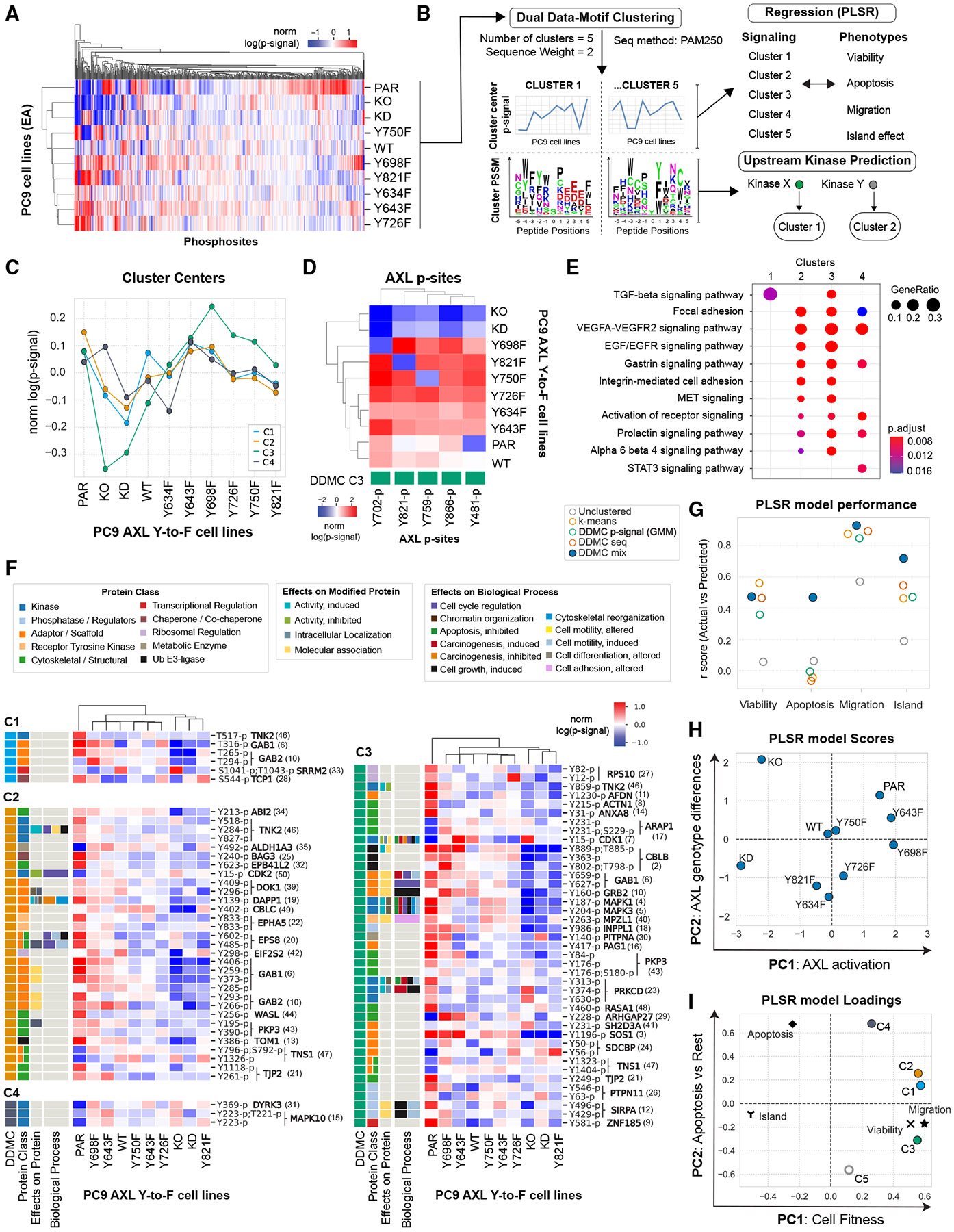
DDMC signaling clusters predict the AXL-mediated oncogenic phenotypes (A) Hierarchical clustering of phosphoproteomic measurements from PC9 AXL Y-to-F cell lines. (B) Computational workflow linking DDMC-derived phosphosite clusters to phenotypic responses by PLSR. (C) Average relative phosphorylation signal of DDMC cluster centers. (D) AXL phosphosite phosphorylation and C3 assignments. (E) Ranked GSEA of DDMC clusters. (F) Hierarchical clustering of the top 50 AXL-regulated proteins, with annotated protein classes and phosphosite effects. (G) PLSR prediction performance across clustering strategies. (H and I) PLSR scores and loadings.

To determine whether variation in downstream signaling could account for these distinct phenotypic outcomes, we employed a previously described mass spectrometry approach^36^ to measure the signaling effect of AXL activation in the PC9 AXL mutant cells during EGFR inhibition. We previously showed that maximal AXL phosphorylation occurs at 5–10 min of AF154 treatment^32^ and, through a detailed time course of response to E,^37^ observed that E leads to rapid loss of proliferative signaling in PC9. This led us to pre-treat PC9 cells with 1 μM of E for 4 h and subsequently treat with EA (1 μM E and 300 ng/mL A) for 10 min prior to lysis. Hierarchical clustering of the phosphoproteomic data showed AXL KO and KD clustered together, as expected, whereas the other cell lines did not display obvious clustering (Figure 2A).

We next applied dual data-motif clustering (DDMC), a computational approach we previously developed to integrate phosphorylation abundance and peptide-sequence information to group phosphosites into signaling clusters.^38^ We then used PLSR to relate these clusters to phenotypic responses and used DDMC-generated cluster sequence motifs to infer candidate upstream kinases^38^ (Figure 2B, supplemental information). Model selection identified five clusters, a sequence weight of 2, and two PLSR components, with combined abundance/sequence models outperforming single-source models (Figures S2A-S2D, see STAR Methods). Notably, models integrating both abundance and sequence information consistently outperformed those relying on a single information source (Figure S2C). Because each PC9 AXL Y-to-F cell line displays variable ectopic AXL expression, it was unclear whether the identified clusters were affected by AXL dosage. We confirmed, through correlation analyses and analytical removal of AXL abundance, that the DDMC clusters reflect distinct, AXL expression-independent phosphoproteomic signaling programs (Figure S3).

The resulting cluster (C) centers grouped the AXL-responsive behaviors. C1, C2, and especially C3 were markedly decreased in PC9 AXL KO and KD cells, with varied phosphorylation signal across Y-to-F mutants. We were particularly interested in C3 because, in addition to a dramatic phosphorylation decrease in AXL KO and KD, it showed an increased abundance in PC9 AXL Y698F—a mutant that phenotypically behaves like PC9 PAR (Figure 1). Conversely, C4 shows an increase in PC9 KO—but not KD—with respect to PAR, and a lower phosphorylation signal in Y634F compared to the rest of clusters (Figure 2C). Cluster 5 had biologically inexplicable signaling trends and so was disregarded (Figure S2E). We next investigated the cluster membership for all AXL phosphorylation sites (phosphosites) and found all of them within C3, consistent with C3 displaying the most dramatic AXL responsiveness (Figures 2C and 2D). The AXL phosphorylation differences (Figure 2D) did not correlate with total or cell surface AXL levels (Figure S1C) suggesting that the variation in signaling was not due to varying ectopic AXL expression.

GSEA revealed distinct functional features for each cluster. Cluster C1 showed strong enrichment for TGF-beta signaling, while C4 was uniquely enriched in a STAT3 signaling signature (Figure 2E). Clusters C2 and C3 shared biological processes associated with the regulation of focal adhesions and the activation of RTK signaling, specifically involving EGFR, VEGFR, and MET. Collectively, these results confirm known AXL-mediated processes, such as downstream EGFR pathway re-activation and focal adhesion regulation,^5,39^ while highlighting less established programs involving TGF-beta and STAT3.

We constructed a STRING network where node size represents the magnitude of AXL phospho-modulation, defined as the cumulative difference in phosphorylation abundance between PC9 PAR and AXL KO cells (Figure S2F). We manually annotated protein classes and phosphosite effects from PhosphoSitePlus for the top 50 AXL-regulated proteins^40^ (Figure 2F). Hierarchical clustering indicated that phosphorylation profiles in AXL KD and AXL Y821F mutant cells were consistently similar to AXL KO cells, and AXL Y698F cells were similar to PC9 PAR, whereas trends in other mutants varied on a case-by-case basis.

Among the kinases most strongly influenced by AXL were Erk1/2 and Cdk1. Erk1 Y187-p and Erk2 Y204-p activate mitogenic and migratory programs, including those implicated in AXL-mediated resistance.^7^ Inhibitory Cdk1 Y15-p and Cdk2 Y15-p were elevated in AXL KO cells relative to PAR cells. Because Y15 phosphorylation suppresses kinase activity and cell cycle progression,^41,42^ AXL signaling likely promotes Y15 dephosphorylation to facilitate mitosis in PC9 cells (Figure 2F).

AXL activation also increased phosphorylation of proximal RTK adaptors, including GAB1, GAB2, and GRB2 Y160-p, which recruit and amplify downstream signaling.^30,43^ In addition, SHP2 (PTPN11) Y546/Y63 and SIRPA Y496/Y529 phosphorylation were higher in PC9 PAR than AXL KO cells. SHP2 is an oncogenic resistance-associated phosphatase, while phosphorylated SIRPA can recruit SHP2.^44^ Finally, many AXL-regulated proteins, including Ack1, Prkcδ, EPS8, WASL, and ACTN1, are linked to cytoskeletal remodeling and migration (Figure 2F).

### DDMC clusters predict AXL-mediated phenotypic responses and identify C1, C2, and C3 as downstream drivers of cell viability and migration

After finding strong correlations between DDMC cluster center, AXL phosphosites, and phenotypic responses (Figures S2K and S2L), we regressed DDMC cluster centers against phenotypic responses using PLSR (Figure 2B). We compared the predictive performance of DDMC (prioritizing both phosphorylation abundance and peptide sequences, “DDMC mix”) against other strategies, including no clustering, k-means, a Gaussian Mixture Model (GMM), and sequence-only DDMC. Only the “DDMC mix” cluster centers accurately predicted all four phenotypes. Conversely, a PLSR model fit directly to the MS data failed, likely due to the high dimensionality of the peptide data relative to the number of cell lines (Figures 2G and 2H).

The resulting PLSR model illustrated the varying capacity of PC9 cell lines to promote proliferation and migration. PC1 corresponded to overall cancer cell fitness (Figures 2H and 2I), mirroring the PCA of AXL phenotypes (Figures 1F and 1G). PC9 PAR cells and mutants Y698F and Y643F associated positively with fitness along PC1, while PC9 AXL KO and KD weighted negatively, associating strongly with E-induced apoptosis and the island effect. PC2 captured variation specific to apoptosis; mutants Y634F and Y821F weighted negatively on this axis, consistent with their exclusive ability to reduce apoptosis (Figures 1C and 2I). Notably, these mutants did not weight on PC1, confirming they do not affect proliferation or migration. Finally, we found that C3 strongly associates with cell migration and proliferation along both PC1 and PC2, whereas C1 and C2 associate with these phenotypes only across PC1 (Figures 2H and 2I). Collectively, these results suggest that AXL activates C1, C2, and especially C3 to promote cell proliferation and migration in the presence of E.

### AXL downstream signaling correlates with poor patient survival and progressive disease in EGFRm LUAD patients

**Figure 3.**
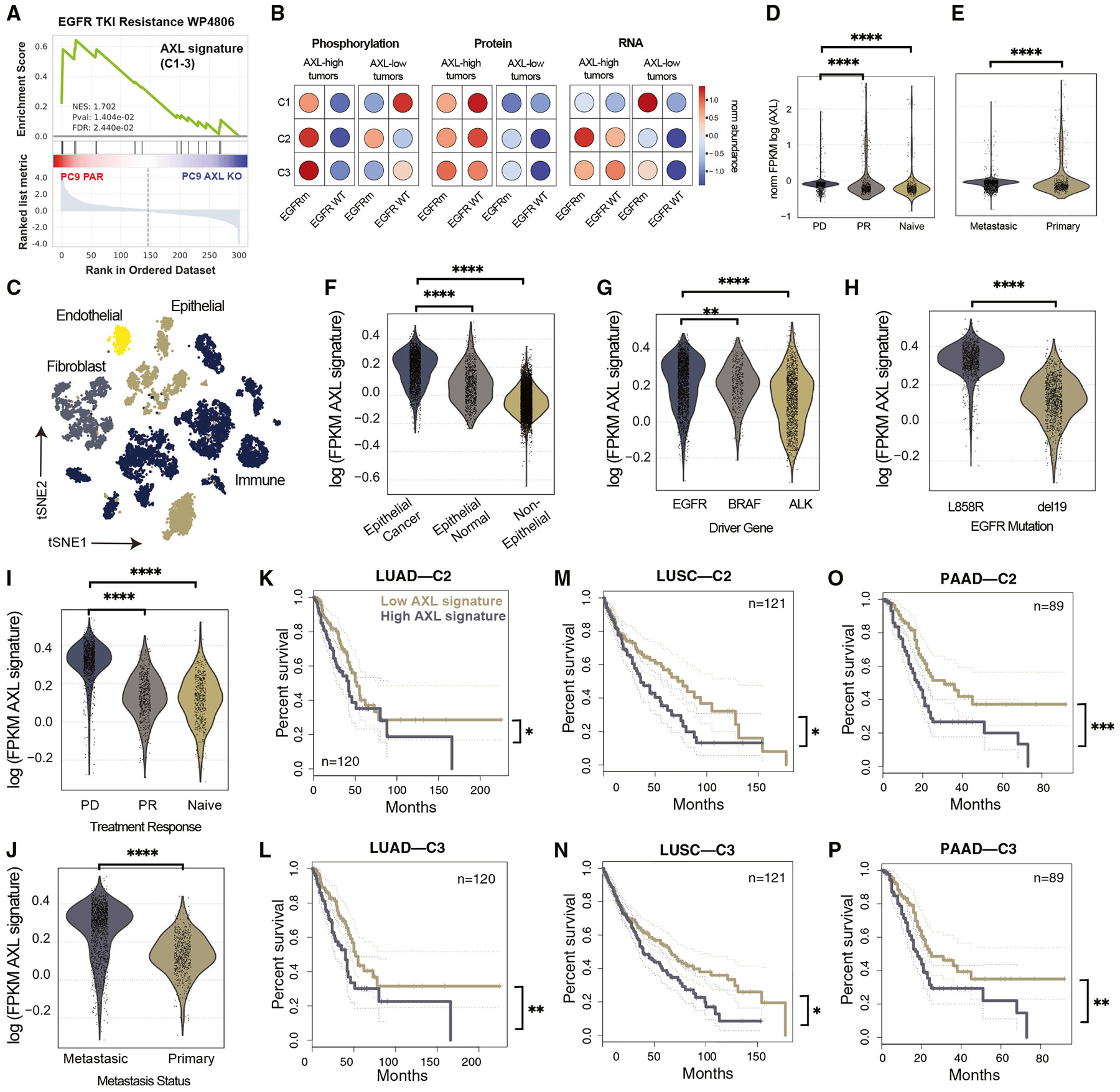
AXL downstream signature based on C2 and C3 is specific to AXL-high EGFRm LUAD tumors, is conserved across omics, and correlates with poor clinical outcomes (A) Ranked GSEA of an EGFR TKI resistance signature using C1–C3 phosphosites ranked by differential phosphorylation between PC9 PAR and AXL KO cells. (B) Phosphorylation, protein, and gene-expression levels of C1–C3 members in LUAD tumors stratified by AXL protein and EGFR genotype. (C) tSNE of LUAD patient samples from Maynard et al. (D and E) AXL expression by treatment response and sampling site. (F–J) AXL signaling score by cell type, driver mutation, EGFR mutation, treatment response, and metastatic status. (K–P) Kaplan-Meier analysis of patients stratified by C2 or C3 expression. Statistical signifcance assessed using log rank tests. * *p* < 0.05, ** *p* < 0.01, *** *p* < 0.001, **** *p* < 0.0001; ns, not significant.

To explore the clinical relevance of the identified signaling and its association with EGFR therapy resistance, we first defined an “AXL downstream signature” by ranking phosphosites in clusters C1, C2, and C3 based on their differential abundance in PC9 PAR versus PC9 AXL KO. GSEA demonstrated that this signature is enriched for EGFR TKI resistance (Figure 3A).

We next utilized proteogenomic data from the NCI’s CPTAC LUAD study to explore the expression of C1-3 in lung cancer patients at the phosphorylation, protein, and transcriptional level.^45^ Stratifying tumors by AXL protein expression and EGFR genotype revealed that all three clusters showed an increased phosphorylation in AXL-high EGFRm tumors specifically, whereas C2 and C3 members were significantly enriched at both the protein and transcript levels in AXL-high tumors irrespective of EGFR genotype. While C1 members showed similar differences in protein and phosphorylation abundance to C2 and C3, C1 gene expression was not elevated in AXL-high tumors (Figure 3B).

Although phosphorylation and transcriptional changes frequently diverge, the transcriptional enrichment of clusters C2 and C3 in AXL-high tumors (Figure 3B) warranted further investigation. Using published single-cell RNA sequencing (scRNA-seq) data, we examined AXL and its downstream signature (C1–3) in EGFRm tumors with reported therapeutic resistance (Figure 3C).^46^ We observed that AXL expression was significantly increased in progressive disease and metastatic tumors (Figures 3C-3E). Similarly, the phosphoproteomic AXL signature was upregulated in L858R EGFRm cancer cells and metastatic tumors compared to normal epithelial cells (Figures 3F-3J). Notably, this upregulation was driven by genes in C2 and C3, but not C1, consistent with our bulk RNA sequencing (RNA-seq) observations (Figures S4 and 3B).

Using The Cancer Genome Atlas (TCGA) data, we observed that higher gene expression of C2 and C3 is associated with significantly reduced overall survival in LUAD, lung squamous cell carcinoma (LUSC), and pancreatic adenocarcinoma (PAAD) (Figures 3K-3P). The enrichment of this signature in PAAD patients with poor outcomes is consistent with the prevalence of mutant EGFR and the implication of AXL in TKI resistance within these tumor types.^6,17,47^

### Dasatinib selectively targets C2 and C3

**Figure 4.**
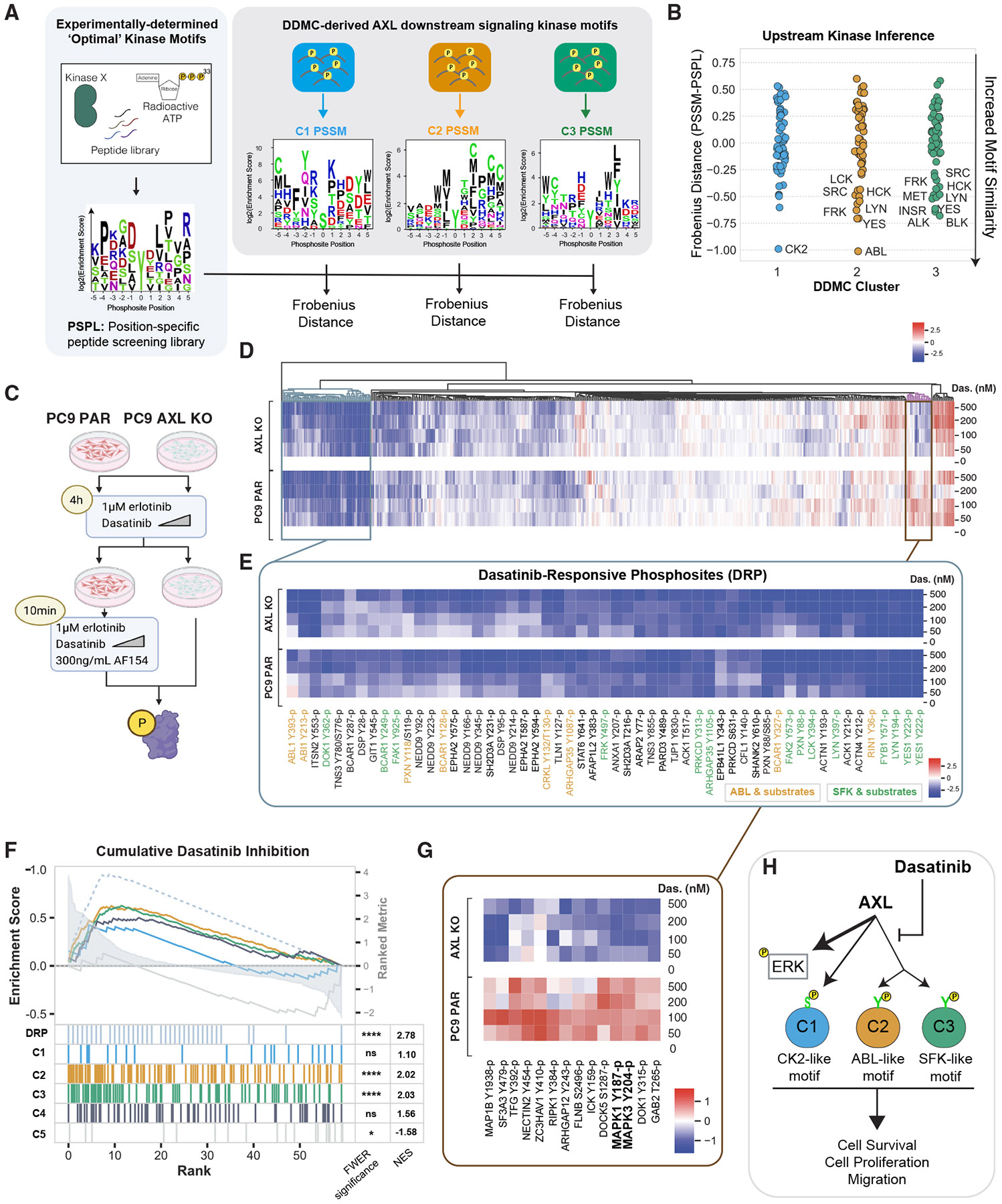
Dasatinib inhibits C2 and C3 (A) Workflow for DDMC-based upstream kinase prediction by comparing cluster PSSMs with published kinase specificity motifs. (B) DDMC upstream kinase predictions. (C) pY mass spectrometry design after erlotinib, dasatinib, and acute AXL activation. (D) Hierarchical clustering of dasatinib-treated PC9 PAR and AXL KO phosphoproteomes. (E) Heatmap of dasatinib-responsive phosphosites. (F) Ranked GSEA comparing dasatinib-responsive phosphosites with DDMC clusters. (G) ERK1/2-containing phosphosite cluster showing AXL-dependent but dasatinib-insensitive phosphorylation. (H) Model of dasatinib effects on AXL downstream signaling.

Based on the correlation between poor prognosis in LUAD patients and the expression of proteins in clusters C2 and C3, we investigated the regulation of these clusters by AXL signaling. We used DDMC to construct position-specific scoring matrices (PSSMs) representing residue frequency for each cluster, comparing them against 60 published kinase motifs to infer upstream regulators (Figures 2B and 4A).^38^ DDMC predicted that CK2, Abl1, and SFK act upstream of C1, C2, and C3, respectively (Figure 4B).

We therefore tested whether dasatinib, albeit a broad-spectrum kinase inhibitor, would selectively inhibit C2 and C3 due to its potent effects on ABL/SFK inhibition and downregulation of cytoskeletal remodeling and migration.^48,49^ PC9 PAR and AXL KO cells were pretreated with E plus increasing dasatinib concentrations before acute AXL activation and pY mass spectrometry (Figure 4C).

Hierarchical clustering revealed a cluster of dasatinib-sensitive peptides, including sites on Abl1, SFK, and downstream targets such as Ack1, Prkcδ, FAK1, and cytoskeletal regulators among others (Figures 4D and 4E). Importantly, these dasatinib-sensitive peptides showed a statistically significant enrichment overlap with C2 and C3, but not C4 or C5 (Figure 4F). We also identified a cluster containing Erk1 Y187-p and Erk2 Y204-p that remained phosphorylated in dasatinib-treated PAR cells but was inhibited in AXL KO cells (Figure 4G), suggesting that AXL regulates Erk activation independently of C2 and C3. Together, these results indicate that dasatinib specifically inhibits C2 and C3 by strongly dephosphorylating upstream kinases including Abl1 and SFKs (Figure 4H).

### A high-throughput bacterial peptide display screen characterizes AXL kinase specificity and identifies FAK1 as a top substrate

**Figure 5.**
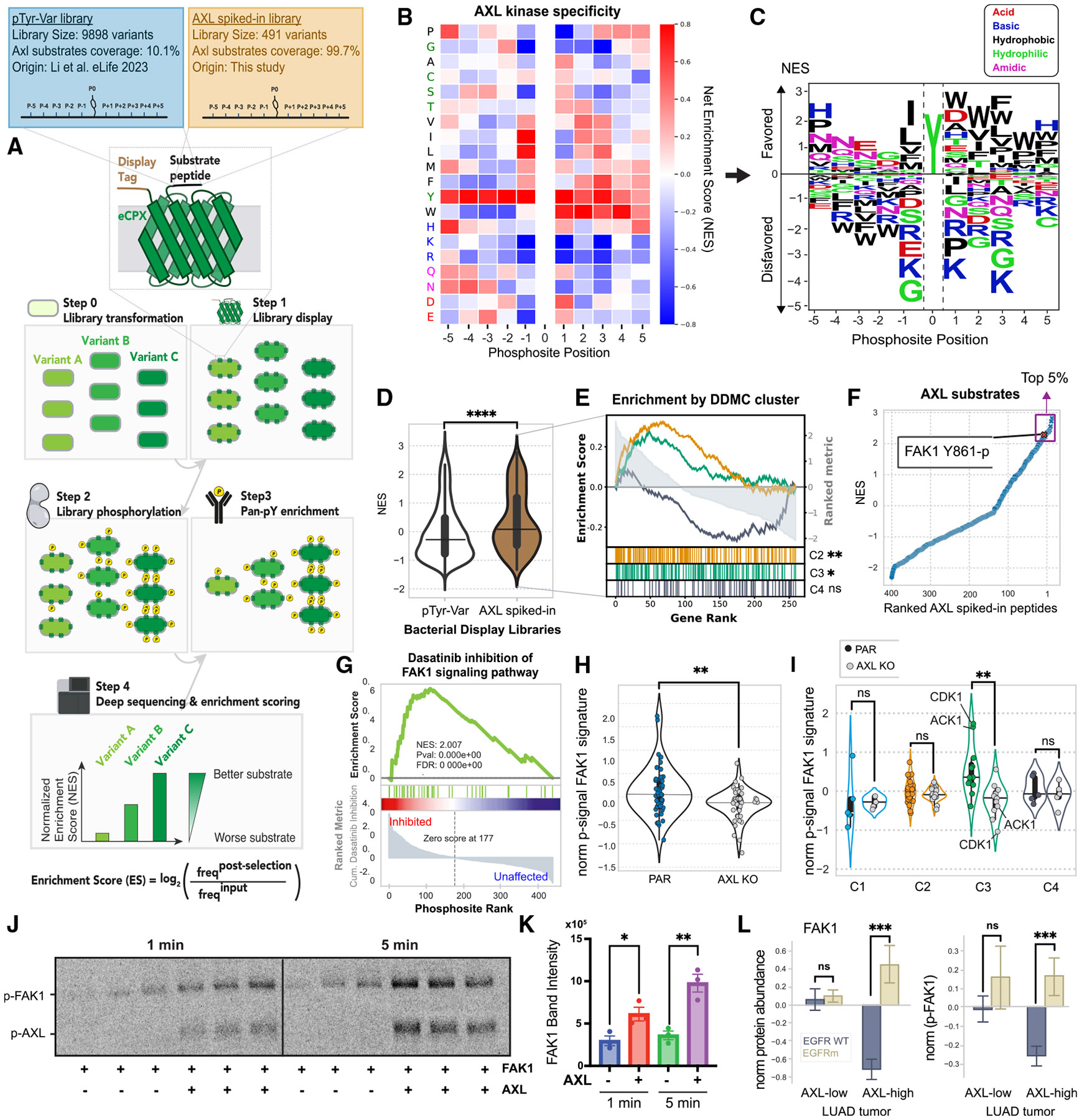
AXL kinase specificity profiling identifies FAK1 as a direct substrate (A) Bacterial peptide-display workflow. (B and C) AXL substrate motif shown as a PSSM heatmap and logo plot. (D) NES distribution of phosphosites present or absent in the AXL phosphoproteomics dataset. (E) Ranked GSEA of AXL MS peptides sorted by NES. (F) Ranked AXL substrates. (G) GSEA of FAK1 pathway phosphosites ranked by dasatinib sensitivity. (H and I) Phosphorylation of FAK1 pathway members by cell line and DDMC cluster. (J and K) *In vitro* kinase assay testing AXL-mediated phosphorylation of full-length FAK1. (L) FAK1 protein and pan-phosphorylation in EGFRm LUAD tumors stratified by AXL abundance. Statistical significance was calculated using Mann-Whitney U rank tests in (D), (E), and (H–K), whereas a Student’s t-test was used in (J). In (J) and (K), the error bars are defined by the error of the mean. * *p* < 0.05, ** *p* < 0.01, *** *p* < 0.001, **** *p* < 0.0001; ns, not significant.

To ascertain which identified phosphosites are directly phosphorylated by AXL, we employed a bacterial peptide display screen.^50^ In this system, a library of peptides is displayed on the surface of *E. coli* via an eCPX receptor, phosphorylated by the kinase of interest, and enriched using a pan-pY antibody (Figure 5A; Figures S5A-S5C). This approach allowed us to simultaneously define AXL’s optimal consensus motif and assess the phosphorylation likelihood of specific peptides from our phosphoproteomics dataset. We screened two libraries: the “pTyr-Var” library containing ~10,000 annotated human tyrosine phosphosites,^50^ and a custom “AXL spiked-in library” containing the 508 pY peptides observed in the unfiltered AXL MS dataset (Figures 5A and S5D).

We computed a PSSM to define the AXL substrate motif, which closely resembled that of the related TAM-family kinase MerTK (Figures 5B and 5C).^51^ AXL exhibited a strong preference for hydrophobic residues at C-terminal positions +1 to +5, but also hydrophilic residues—particularly aspartate and histidine—at +1. At the N terminus, AXL favored asparagine at positions −5 through −3 and displayed a strong preference for isoleucine or leucine at −1, a common hallmark of tyrosine kinases.^52^ Conversely, AXL disfavored basic residues like lysine and arginine. Side-by-side comparison of the libraries revealed that phosphosites from our dataset were preferentially phosphorylated by AXL relative to the general phosphoproteome (Figure 5D). Furthermore, high-scoring substrates were enriched in clusters C2 and C3, but not C4 (Figure 5E). Subcellular localization analysis indicated that preferred AXL substrates are associated with the cell membrane (Figure S5H).

While we did not detect SFK or Abl1 peptides as direct AXL substrates, the activating FAK1 phosphosite Y861 was among the top 5% AXL-regulated sites (Figure 5F). FAK1, a key regulator of SFKs, focal adhesions, and migration, has been linked to malignancy and metastasis, and a prior study showed that a FAK1 pathway signature in EMT-mediated E-resistant lung cancer cells was reversed by dasatinib.^49,53,54^ Many proteins that our model identified as mediating AXL-driven migration and proliferation overlapped this FAK1 pathway signature (Figures 2F and 2I), and our AXL downstream signature was similarly depleted by dasatinib, mirroring the effects on clusters C2 and C3 (Figure 5G). AXL-activated PC9 cells exhibited increased phosphorylation of FAK1 pathway components compared with AXL KO cells, driven mainly by C3, with Cdk1 and Ack1 showing the strongest activation (Figures 5H and 5I). In an *in vitro* kinase assay with full-length FAK1 and [γ-^33^P] ATP, the presence of AXL markedly increased FAK1 phosphorylation beyond its modest autophosphorylation (Figures 5J and 5K), confirming FAK1 as a direct AXL substrate *in vitro*. Consistent with these findings, EGFRm AXL-high LUAD tumors showed elevated FAK1 expression and phosphorylation (Figure 5L).

Among the top-scoring AXL substrates, our *in vitro* phosphorylation screen identified several known AXL substrates, such as Ack1 (TNK2), SHP-2, CTNND1, and PEAK1^19,20,55^ as well as less established interactors including the cytoskeletal-remodeling proteins TJP2, BCAR1, TNS1, ANXA2/5, TWF1, ITSN2, the kinase DYRK1A/3, and the E3-ubiquitin ligases CBLB and CBLC (Figure S5I). Six different NEDD9 phosphosites were among the top 10% AXL substrates, consistent with the GSEA enrichment of C2 and C3 and previous studies describing the role of AXL in regulating focal adhesions and migration through this interaction (Figures 2E and S4I).^19^

### AXL acts upstream of YAP signaling, which is upregulated in RTK inhibitor-resistant EGFRm LUAD tumors

**Figure 6.**
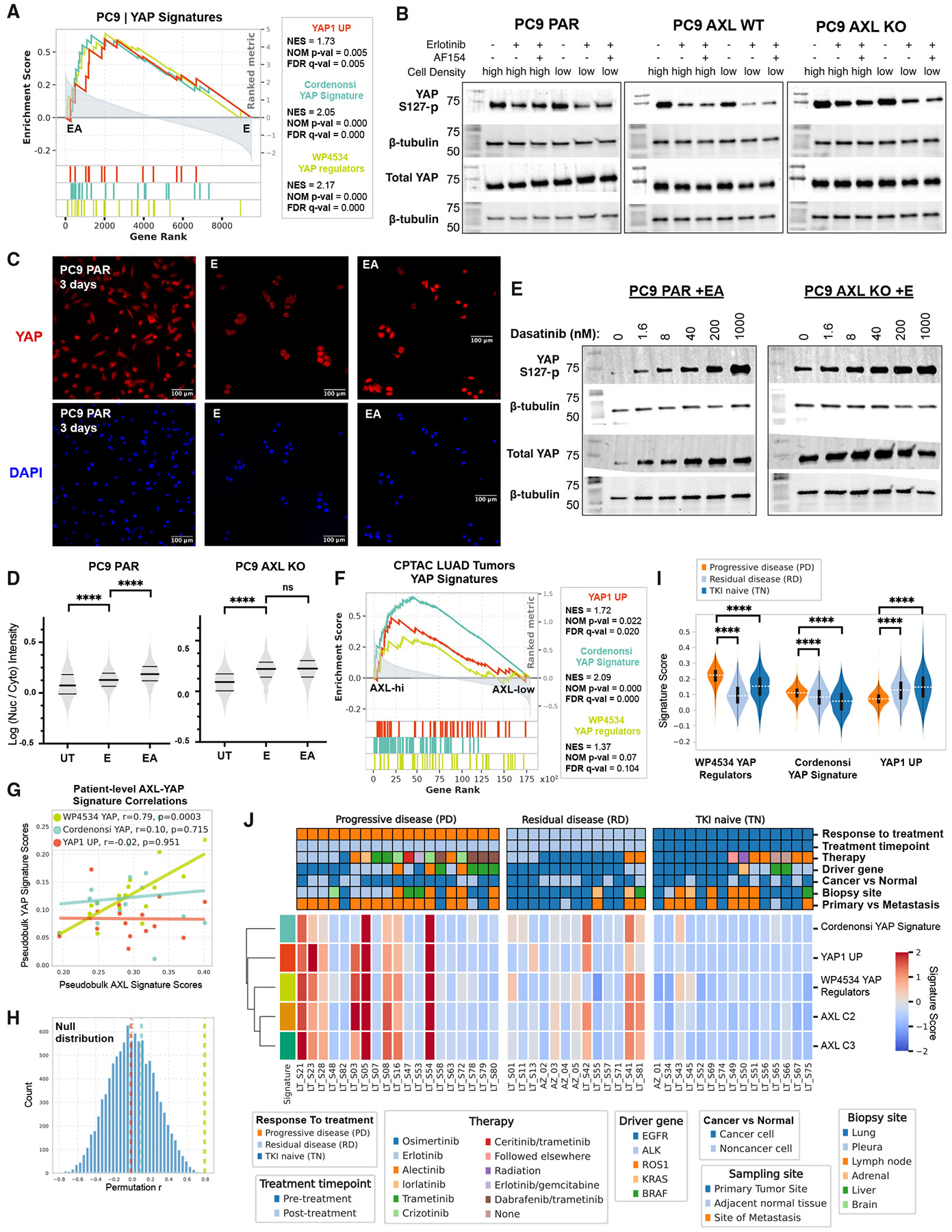
The AXL-YAP signaling axis is enriched in progressive EGFRm LUAD patients (A) GSEA of RNA-seq from AXL Y-to-F mutants ranked by PCA scores, showing YAP signature enrichment in EA-versus E-treated cells. (B) Total and S127-p YAP in PC9 PAR, AXL WT, and AXL KO cells under density and treatment perturbations. (C and D) YAP immunofluorescence and nuclear-localization quantification after E or EA treatment. Scale bars represent 100 μm. (E) Total and S127-p YAP after EA plus increasing dasatinib. (F) GSEA of YAP signatures in AXL-high versus AXL-low LUAD tumors. (G and H) Patient-level correlations between AXL and YAP signatures. (I) YAP signature scores in EGFRm LUAD tumors grouped by treatment response. (J) Patient-level heatmap of AXL- and YAP-related signatures across clinical groups. Correlations between patient-level signature scores in (G) and (H) were evaluated using Pearson correlation coefficients, with significance estimated by permutation testing using 10,000 label permutations. * *p* < 0.05, ** *p* < 0.01, *** *p* < 0.001, **** *p* < 0.0001; ns, not significant.

To characterize the transcriptional changes driving AXL-mediated phenotypes, we analyzed RNA-seq data from PC9 AXL Y-to-F cell lines treated with E or EA. PCA indicated that PC1 captures AXL activation (Figure S6A). GSEA based on PC1 scores identified three YAP signatures: “YAP1_UP” and “Cordenonsi,” which represent transcriptional programs downstream of YAP activation, and “WP4534,” a curated network mapping YAP/TAZ regulation during mechanotransduction (Figures 6A and S6B).^56,57^ Given that AXL regulates mechanosensing components like focal adhesions (Figures 2E, 2F, and S5H), and that the upstream and downstream signatures share minimal overlap (Figure S6C), these data suggest AXL influences YAP regulation.^19,54^ This aligns with our upstream kinase predictions identifying Abl1 and SFKs—known drivers of YAP nuclear translocation—as AXL effectors.^58-60^

To validate AXL as a key upstream regulator of the YAP pathway, we assessed the inhibitory YAP phosphorylation site S127.^58^ While S127-p decreased at low cell density in response to E and EA, AXL KO cells maintained significantly higher S127-p levels compared to PAR or WT cells, indicating AXL is required for optimal YAP activation (Figure 6B). We treated cells with E or EA for 3 days to observe YAP localization in cancer cells in the presence of inhibitor. PC9 PAR cells treated with EA displayed significantly higher nuclear YAP than cells treated only with E (Figures 6C and 6D). Consistent with the western blots of YAP S127-p, we observed AXL-independent, erlotinib-induced YAP nuclear translocation (Figures 6B-6D; Figure S6D). Dasatinib increased S127-p abundance, an effect also seen by AXL KO (Figure 6E). Dasatinib also strongly inhibited AXL protein expression, supporting prior evidence of a feedback loop wherein YAP transcriptionally regulates AXL (Figure S6E).^61-63^

We next investigated the AXL-YAP axis in human lung tumors using the CPTAC proteogenomic data from 110 LUAD patients (Figure 3B). AXL-high tumors exhibited elevated EMT markers (Figure S6F) and significant enrichment of YAP downstream signatures (Cordenonsi and YAP1_UP), with weaker enrichment of the upstream WP4534 signature (Figure 6F). Conversely, analysis of scRNA-seq data revealed a strong correlation between the AXL downstream signature and the YAP upstream regulator signature (WP4534), but not the YAP downstream signatures (Figures 6G and 6H), despite minimal gene overlap (Figure S6C).

Finally, we assessed associations with therapeutic response. The WP4534 and Cordenonsi signatures were significantly upregulated in EGFRm tumors that did not respond to treatment, whereas the YAP1_UP signature showed the opposite trend (Figure 6I). Pseudobulk scoring linked these signatures to metastatic progressive disease, consistent with their role in regulating focal adhesions and migration (Figure 6J). Notably, elevated AXL and YAP signatures were observed in ALK- and ROS1-driven cases as well as EGFR-driven tumors. Collectively, these results suggest that the AXL-YAP signaling axis-identified *in vitro* is clinically relevant and correlates with erlotinib resistance in LUAD (Figures 3, 6F-6J, and S6F).

### Inhibition of AXL downstream signaling and YAP eliminates DTP cells

**Figure 7.**
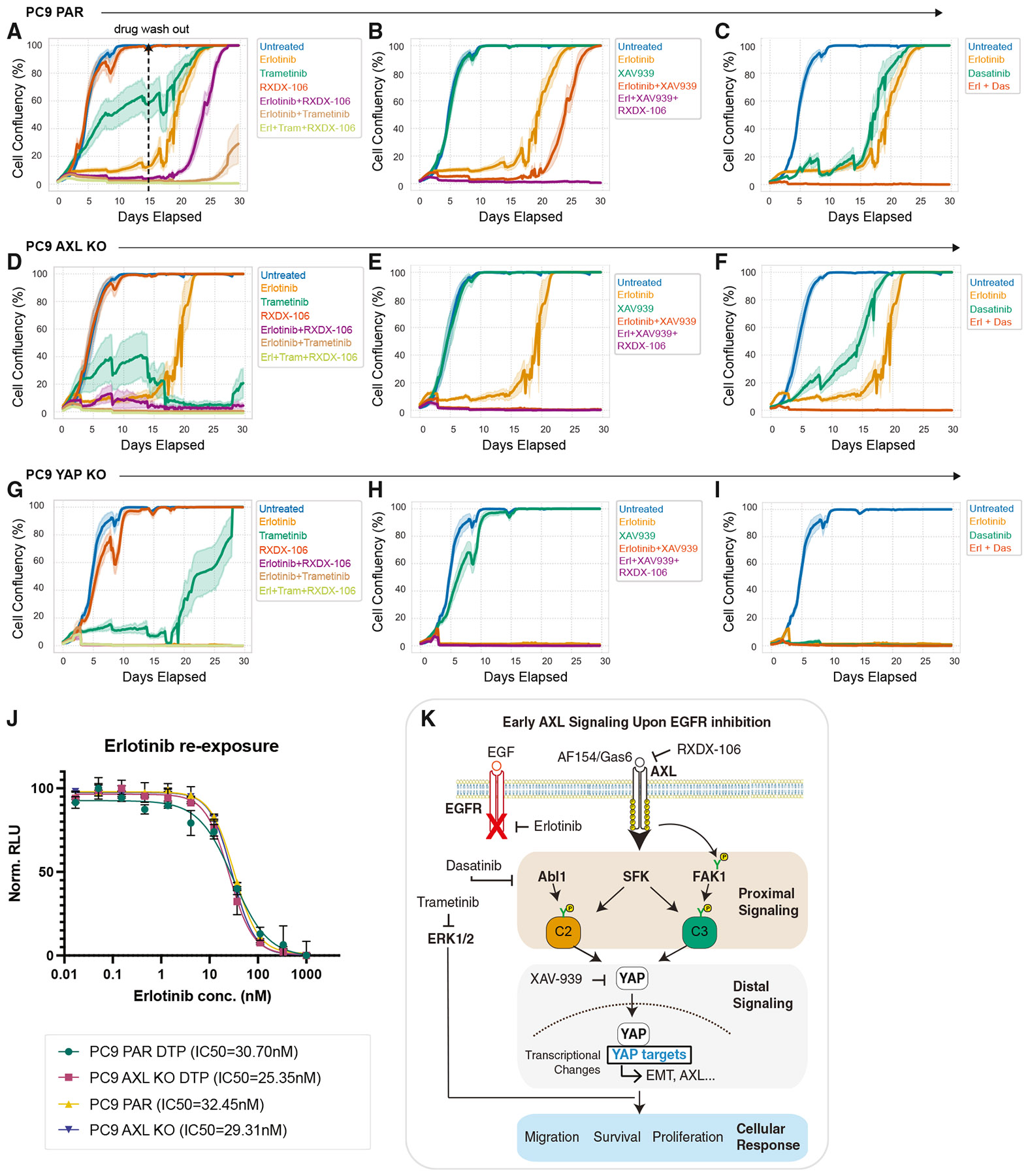
Targeting AXL downstream signaling inhibits drug-tolerant persister cells (A–I) Long-term viability and regrowth assays of PC9 PAR, AXL KO, and YAP KO cells treated with erlotinib alone or combined with inhibitors of AXL, Erk1/2, SFK/Abl1/FAK1, or YAP. (J) Erlotinib retreatment sensitivity of drug-tolerant persister cells compared with freshly thawed controls. (K) Model of early AXL-driven signaling after EGFR inhibition.

Prior studies link YAP activation and FAK1 signaling to the development of drug resistance and drug-tolerant persister (DTP) cells during EGFR-targeted therapy.^61,64-68^ Since our phosphoproteomics data indicate that AXL activates FAK1 (Figures 5F-5K), we investigated AXL signaling in DTP emergence. We treated PC9 PAR, AXL KO, and YAP KO cells for 15 days with E alongside inhibitors for AXL (RXDX-106), Erk1/2 (trametinib), SFK/Abl1/FAK1 (dasatinib), or YAP (XAV-939^65,69,70^). While combining E with AXL inhibition modestly delayed DTP emergence, dual EGFR, and Erk1/2 blockade proved more inhibitory. However, triple inhibition of EGFR and AXL alongside either YAP or Erk1/2 completely blocked persister cell emergence (Figures 7A and 7B). Similarly, combining E with dasatinib, which disrupts both AXL and YAP activity, prevented regrowth (Figure 7C). These trends persisted in AXL KO cells (Figures 7D-7F), whereas PC9 YAP KO cells failed to develop resistance under most conditions, underscoring YAP’s critical survival role (Figures 7G-7I). The combination of RXDX-106 and E showed a greater inhibitory effect than E alone, indicating AXL-independent effects. We confirmed the distinct DTP phenotype by demonstrating that surviving PC9 PAR and AXL KO cells remained fully sensitive to E upon retreatment (Figure 7J).

Based on these findings, we propose a signaling model in which AXL activation drives resistance (Figure 7K). Our modeling and specificity screening indicate that AXL engages adapters including GAB1/2 and SOS1, cytoskeletal proteins, and kinases such as FAK1 (Figures 2 and 5). We focused on phosphotyrosine clusters C2 and C3, which correlated with malignancy and displayed kinase motifs for SFK and Abl1 (Figure 4B; Figures S2H-S2J). Dasatinib selectively decreased AXL-mediated phosphorylation of these clusters (Figures 4B and 4F). We validated FAK1 as a top AXL substrate enriched in cluster C3 and observed that dasatinib substantially inhibited the FAK1 pathway signature (Figures 5F-5I). Downstream, AXL activation upregulated YAP transcriptional signatures and induced YAP nuclear translocation (Figures 6A-6D; Figure S6D). Consequently, targeting this axis via dasatinib or triple inhibition of EGFR, AXL, and YAP/Erk1/2 effectively blocks the emergence of drug-tolerant persisters (Figures 7A-7I).

## DISCUSSION

Reactivation of oncogenic pathways through RTKs not targeted by therapy, known as RTK bypass signaling, is a common resistance mechanism.^71-74^ However, the complexity of RTK cross talk and pathway interdependency has hindered the identification of the specific signaling networks that drive this resistance.^37,71^ To address this, we employed a combined computational and experimental approach using a panel of PC9 AXL tyrosine-to-phenylalanine (Y-to-F) mutants. Because PC9 cells express surface AXL but maintain low baseline activation, this system allowed us to isolate the earliest signaling perturbations and phenotypic changes specifically driven by AXL activation following EGFR inhibition, rather than modeling long-term acquired resistance. By applying multivariate modeling to this dataset, we deconvolved the network-level changes across mutants and linked specific phosphoproteomic signatures to cell fitness and migration.

We identified three phosphosite clusters (C1, C2, and C3) that define an “AXL downstream signature.” Crucially, clusters C2 and C3—but not C1—correlated with poor treatment response, metastasis, and overall survival in EGFRm LUAD patients. This signature was significantly enriched in AXL-high tumors, particularly in EGFRm samples compared to WT. Although our study focused on AXL, we observed that these downstream signals were also elevated in subsets of ALK- and reactive oxygen species (ROS)-positive patient samples. This suggests that the identified bypass machinery, particularly the interplay between AXL and downstream effectors, may be conserved across various TKI-resistant tumors driven by RTKs other than EGFR. Future implementation of this methodology across a wider panel of receptors could further elucidate whether this pathway activation is a universal mechanism of RTK bypass resistance.

Mechanistically, our data clarifies the relationship between AXL and the Hippo pathway effector YAP.^59,60,64,75^ While YAP is known to upregulate AXL, our findings indicate that AXL acts upstream to drive YAP activation in a feed-forward loop.^61,62,66^ Dasatinib treatment selectively inhibited C2 and C3 and blocked YAP activation, suggesting these clusters function upstream of the transcription factor. While C2 and C3 are enriched for kinase motifs favored by SFK and Abl1, and previous reports have indicated that these kinases can be phosphorylated by AXL,^18,31,76^ our bacterial display specificity screen revealed that AXL does not preferentially phosphorylate these kinases directly. Instead, we identified FAK1, a known regulator of SFK signaling,^49,77^ and the Abl1 activator RIN1,^78-80^ as top-ranked AXL substrates. This suggests that AXL likely activates SFK and Abl1 signaling indirectly through intermediates like FAK1 to sustain drug-tolerant persister cell survival.^68^

Finally, our results highlight a critical signaling plasticity that complicates therapeutic intervention. Resistant cells to single-agent osimertinib can emerge through a variety of mechanisms, including Erk1/2 reactivation or YAP activation, whereas YAP activation becomes the dominant resistance mechanism of cells overcoming combined EGFR and MEK inhibition.^65^ We similarly observed that cancer cells can toggle between Erk1/2 and YAP signaling to overcome EGFR inhibition depending on the context. For instance, while dasatinib successfully inhibited the AXL-YAP axis (specifically C2 and C3), it paradoxically promoted AXL-dependent Erk1/2 reactivation. Our long-term viability assays confirm that preventing the emergence of DTP cells requires additionally blocking downstream effectors, such as YAP or its upstream kinases, whereas combined inhibition of EGFR and AXL alone only delays resistance. Collectively, these data suggest that systematically profiling Y-to-F mutants can identify key bypass mechanisms and that concomitantly targeting downstream signaling networks provides superior therapeutic efficacy to inhibiting RTKs alone.

### Limitations of the study

AXL-transduced PC9 cell lines expressed about 25% of the AXL found on the surface of its PAR counterpart, but around 4-fold more receptor overall (Figure S1). We used phosphoproteomic and phenotypic measurements from these cell lines to hypothesize about the key signaling pathways activated by AXL. We then used proteogenomic datasets of LUAD patients, along with experimental validations on PC9 PAR and AXL KO cells, to confirm the signaling changes identified by our model. Thus, although the varying AXL expression across cell lines is a limitation, the insights from this study are considered in the context of the follow-on experimental and computational validation. Notably, all experiments were conducted using a single, representative EGFRm NSCLC cell line (PC9). As explained previously, this line was selected for its unique capacity to accurately model AXL-mediated effects following EGFR inhibition. Although a link has been established between AXL downstream signaling and erlotinib resistance in EGFRm NSCLC patient samples, further research is required to formally evaluate the generalizability of this signaling architecture in AXL- and RTK-mediated bypass resistance in lung cancer.

## STAR★METHODS

### EXPERIMENTAL MODEL AND STUDY PARTICIPANT DETAILS

PC9 cells were acquired from Sigma Aldrich (90071810-1VL). This cell line is part of the European Collection of Authenticated Cell Cultures (ECACC), originally was derived from a 45-year-old male of Chinese and Japanese background, and was derived from a metastatic lymph node site. As work was performed in the male-derived PC9 line and its derivatives, the influence of sex on the findings could not be evaluated. Using this parental cell line, we generated a panel of 9 additional PC9 derivatives: PC9 AXL KO, PC9 WT, PC9 AXL Y634F, PC9 AXL Y643F, PC9 AXL Y698F, PC9 AXL Y728F, PC9 AXL Y750F, and PC9 AXL Y821F. Cell lines were authenticated using mutational information derived from our RNA sequencing, and were tested for mycoplasma contamination periodically, at least yearly. Please see the methods section “generation of PC9 AXL Y-to-F mutant cell lines” below for additional information.

### METHOD DETAILS

#### Generation of PC9 AXL Y-to-F mutant cell lines

The PC9 AXL KO cell line was generated by transfecting cells with a CRISPR/Cas9 and GFP vector containing a gRNA targeting the AXL kinase domain. The gRNA sequence, cell culturing, and sorting methods have been previously described.^37^ Plasmids containing the AXL phosphosite mutations were generated from an AXL-IRES-Puro vector (Addgene #65627) using site-directed mutagenesis. Each mutant was then inserted into a lentiviral vector with a puromycin resistance marker (Addgene #17448).

For viral packaging, HEK 293T cells were seeded at 4.5 × 10^6^ per 10 cm dish. After 24 h, the lentiviral AXL expression vector, VSV-G envelope vector, and packaging vector (Addgene #12259 and #12260, respectively) were combined in a 10:1:10 mass ratio and diluted in Opti-MEM (Thermo Fisher Scientific). TransIT-LT1 (Mirus Bio) was added dropwise, and the solution was mixed gently by swirling and incubated at room temperature for 20 min. The solution was then added dropwise to the cells. After 18 h, the transfection media was replaced with media supplemented with 1% BSA fraction V (Thermo Fisher Scientific). Cells were incubated for 24 h, after which the virus-containing media was removed and stored at 4°C. The media was replaced, and the cells incubated for a further 24 h to generate a second batch of viral media. The harvested batches were then pooled, filtered through a 0.45 μm PVDF membrane to remove packaging cells, and flash-frozen followed by storage at −80°C until use.

PC9 AXL KO cells were seeded at 1.5 × 10^5^ cells per well with antibiotic-free media in a 6-well plate and incubated for 24 h. The cells were then infected with viral particles in antibiotic-free media supplemented with polybrene (MilliporeSigma). After 18 h, the media was replaced with fresh, antibiotic-free media. Cells were observed for a GFP-positive population and then passaged into a 10-cm plate until confluent. The virally transduced cells were then sorted based on GFP expression using a BD FACSAria cell sorter. The mutant cell populations were subcultured for later experiments.

#### Cell viability and apoptosis assays

Cells were seeded in a 96-well plate at a density of 1.0 × 10^3^ cells per well. After 24 h, treatments were added in media containing 300 nM YOYO-3 (Thermo Fisher Scientific). Cells were cultured and imaged every 3 h using an IncuCyte S3 (Essen Bioscience) at 20× magnification, with 9 images per well. The phase, green, and red channels were manually thresholded and then analyzed by IncuCyte S3 software (Essen Bioscience) to determine cell counts and the fraction of area covered.

#### Cell migration assay

96-well IncuCyte ImageLock plates (Essen Bioscience) were coated with a Collagen I solution (Thermo Fisher Scientific), washed twice, and then seeded with 4 × 10^4^ cells per well. After a 4 h incubation, cells were wounded using the IncuCyte WoundMaker, washed twice to remove detached cells, and then treated with respective conditions. Images of the center of the wound were taken every 2 h at a magnification of 10×, one image per well. The phase, green, and red channels were manually thresholded and then analyzed by IncuCyte S3 software (Essen Bioscience) to determine migration measurements.

#### Cell island effect

Phase contrast images used for cell island measurements were taken from image sets gathered in the cell viability assay. For endpoint readings, images from the 48-h post-treatment time point were used. Representative images were selected across experimental replicates. Images were opened in ImageJ, and the center of each cell was manually marked. Dead cells, identified using YOYO-3 based fluorescence, were not marked. The 2D coordinates of all cell centers in each image were then exported for analysis. The amount of clustering in each image was then measured by applying Ripley’s K function to the set of coordinates. The implementation of Ripley’s K function used was taken from the astropy Python package.^48^

#### Preparation of cell lysates for mass spectrometry

Cell lines were grown to confluence in 10 cm dishes over 72–96 h, washed, and treated by adding media containing 1 μM erlotinib. Cells were incubated for 4 h at 37°C and then additionally treated with media containing 1 μM erlotinib and 300 ng/mL AXL activating antibody for 10 min. The cells were then immediately placed on ice, washed with ice-cold phosphate-buffered saline, and lysed with cold 8 M urea containing Phosphatase Inhibitor Cocktail I and Protease Inhibitor Cocktail I (Boston BioProducts). The lysates were centrifuged at 20,000 x g and 4°C to pellet cell debris, and the supernatants were removed and stored at −80°C. A bicinchoninic acid (BCA) protein concentration assay (Pierce) was performed according to the manufacturer’s protocol to estimate the protein concentration in each lysate. Cell lysates were reduced with 10 mM DTT for 1 h at 56°C, alkylated with 55 mM iodoacetamide for 1 h at RT shielded from light, and diluted 5-fold with 100 mM ammonium acetate, pH 8.9, before trypsin (Promega) was added (20:1 protein:enzyme ratio) for overnight digestion at RT. The resulting solutions were acidified with 1 mL of acetic acid (HOAc) and loaded onto C18 Sep-Pak Plus Cartridges (Waters), rinsed with 10 mL of 0.1% HOAc, and eluted with 10 mL of 40% Acetonitrile (MeCN)/0.1% HOAc. Peptides were divided into 200 aliquots, and sample volume was reduced using a vacuum centrifuge (Thermo) before lyophilization to dryness for storage at −80°C. TMT labeling for multiplexed analysis was performed according to the manufacturer’s protocol. Samples, each containing ~200 μg of peptides, were resuspended in 35 μL HEPES (pH 8.5), vortexed, and spun down at 13,400 rpm for 1 min. 400 μg of a given channel of TMT10plex (Thermo) in anhydrous MeCN was added per sample. Samples were shaken at 400 rpm for 1 h, after which the labeling reaction was quenched using 5% hydroxylamine (50%, Thermo). After another 15 min of shaking, all samples were combined using the same pipette tip to minimize sample loss, and sample aliquots were washed twice with 40 μL of 25% MeCN/0.1% HOAc, which was added to the collection tube to improve yield. Sample volume was reduced using a vacuum centrifuge and then lyophilized to dryness for storage at −80°C.

#### Phosphopeptide enrichment

Immunoprecipitation (IP) and IMAC were used sequentially to enrich samples for phosphotyrosine-containing peptides. TMT-labeled samples were incubated in IP buffer consisting of 1% Nonidet P-40 with protein G agarose beads conjugated to 24 μg of 4G10 V312 IgG and 6 μg of PT-66 (P3300, Sigma) overnight at 4°C. Peptides were eluted with 25 μL of 0.2% trifluoroacetic acid for 10 min at room temperature; this elution was performed twice to improve yield. Eluted peptides were subjected to phosphopeptide enrichment using immobilized metal affinity chromatography (IMAC)-based Fe-NTA spin column to reduce non-specific, non-phosphorylated peptide background. A High-Select Fe-NTA enrichment kit (Pierce) was used according to the manufacturer’s instructions with the following modifications. Eluted peptides from IP were incubated with Fe-NTA beads containing 25 μL binding washing buffer for 30 min. Peptides were eluted twice with 20 mL of elution buffer into a 1.7 mL microcentrifuge tube. The eluates were concentrated in a SpeedVac until approximately 1 μL of sample remained, and then resuspended in 10 μL of 5% acetonitrile in 0.1% formic acid. Samples were loaded directly onto an in-house constructed fused silica capillary column (50 μm inner diameter (ID) x 10 cm) packed with 5 μm C18 beads (YMC gel, ODS-AQ, AQ12S05) and with an integrated electrospray ionization tip (~2 μm tip ID).

#### LC-MS/MS analysis

LC-MS/MS of pTyr peptides was performed on an Agilent 1260 LC system coupled to a Q Exactive HF-X mass spectrometer (Thermo Fisher Scientific). Peptides were separated using a 140-min gradient with 70% acetonitrile in 0.2 mol/L acetic acid at a flow rate of 0.2 mL/min and an approximate split flow of 20 nL/min. The mass spectrometer was operated in data-dependent acquisition with the following settings for MS1 scans: m/z range: 350 to 2,000; resolution: 60,000; AGC target: 3 × 10^6^; maximum injection time (maxIT): 50 ms. The top 15 most abundant ions were isolated and fragmented by higher energy collision-induced dissociation with the following settings: resolution: 60,000; AGC target: 1 × 10^5^; maxIT: 350 ms; isolation width: 0.4 m/z; collisional energy (CE): 33%; dynamic exclusion: 20 s. Crude peptide analysis was performed on a Q Exactive Plus mass spectrometer to correct for small variations in peptide loadings across the TMT channels. Approximately 30 ng of the supernatant from pTyr IP was loaded onto an in-house packed precolumn (100 μm ID x 10 cm) packed with 10 mm C18 beads (YMC gel, ODS-A, AA12S11) and analyzed with a 70-min LC gradient. MS1 scans were performed at the following settings: m/z range: 350 to 2,000; resolution: 70,000; AGC target: 3 × 10^6^; maxIT: 50 ms. The top 10 most abundant ions were isolated and fragmented with a collision energy (CE) of 33% at a resolution of 35,000.

#### Peptide identification and quantification

Mass spectra were processed using Proteome Discoverer, version 2.5 (Thermo Fisher Scientific), and searched against the human SwissProt database with Mascot, version 2.4 (MatrixScience, RRID:SCR_014322). MS/MS spectra were searched with a mass tolerance of 10 ppm for precursor ions and 20 mmu for fragment ions. Cysteine carbamidomethylation, TMT-labeled lysine, and TMT-labeled peptide N-termini were set as fixed modifications. Oxidation of methionine and phosphorylation of serine, threonine, and tyrosine were searched as dynamic modifications. TMT reporter quantification was extracted and isotope corrected in Proteome Discoverer. Peptide spectrum matches (PSM) were filtered according to the following parameters: rank = 1, Mascot ion score >15, isolation interference <40%, and average TMT signal >1,000. Peptides with missing values across any channel were filtered out.

#### Preprocessing of phosphoproteomic data

We performed three biological replicates for phosphoproteomic analysis of PC9 AXL Y-to-F mutants treated with EA. The three datasets were concatenated, mean-centered across cell lines, and log2-transformed. To exclude phosphosites with unreliable measurements across replicates, all recurrent phosphosites in two or three biological replicates were identified. For those appearing in two biological replicates, peptides with a Pearson correlation coefficient less than 0.55 were filtered out; for those appearing in all three biological replicates, peptides with standard deviations of 0.5 or more were discarded. To exclude peptides with minimal variation across cell lines, we filtered out phosphosites showing less than a 0.5-fold change between the maximum and minimum values in each cell line. Overlapping peptides were then averaged across replicates. The resulting preprocessed phosphoproteomic dataset was then fit using DDMC.^50^

#### Prediction of AXL-mediated phenotypes using PLSR

To predict AXL-mediated phenotypes, a 4-component PLSR model (scikit-learn) was built using the DDMC cluster centers. To assess the predictive performance of PLSR, leave-one-out cross-validation was applied. The model was trained using the paired cluster centers and phenotypic measurements in all cell lines except one. The cluster centers of that remaining cell line were then used to predict its phenotypic measurements, and a mean squared error between the predicted and actual values per phenotype was computed. This process was repeated across all cell lines to obtain a final predictive correlation per phenotype. To benchmark the ability of PLSR to predict phenotypes using the DDMC clusters, we also fit PLSR using the unclustered phosphoproteomic data directly, k-means clustering, a Gaussian Mixture Model (GMM), DDMC using only peptide sequence information, or the selected DDMC model combining phosphorylation and sequence information (referred to as DDMC mix). All clustering methods were used with 5 clusters, and the number of PLSR components was optimized for each case.

#### Hyperparameter selection

The preprocessed phosphoproteomic dataset was fit to DDMC using 5 clusters, a sequence weight of 2, and 2 PLSR components. This hyperparameter combination was determined via an exhaustive hyperparameter search (Figures S2A-S2D). We optimized hyperparameters—specifically cluster number, sequence weight, and PLSR components—by evaluating the model’s ability to predict phenotypes. Using scikit-learn’s GridSearch, we assessed the pipeline model—comprised of DDMC and PLSR—to predict AXL-mediated phenotypes using various hyperparameter combinations. These included 2 to 15 clusters, sequence weights of 0, 1, 2, 3, 5, 10, 50, 100, or 500, and 1 to 4 PLSR components. A sequence weight of 0 uses only phosphorylation abundance for peptide clustering; a weight of 500 primarily uses sequence information, and intermediate values combine both information sources. After assessing the interpretability of the top 5% of performing models, we selected a final model consisting of five clusters, a sequence weight of 2, and two PLSR components (Figures S2A-S2D).

#### RNA-seq sample preparation and sequencing

To generate the RNA-seq data, 300,000 cells of each PC9 AXL Y-to-F mutant cell line were seeded in 100 mm dishes. The next day, each dish was treated with E or EA, and after 24 h, cells were lysed with RIPA, and RNA was extracted using the RNeasy Mini Kit (Qiagen). RNA sequencing and read alignment were performed by Novogene. Any genes with less than 10 TPM were filtered out. Ranked or standard GSEA was implemented in Python using the package gseapy.^81^

#### Clinical data and analysis

Bulk RNA-seq, proteomic, and phosphoproteomic data of LUAD patients was obtained from the CPTAC LUAD study.^45^ scRNA-seq of LUAD patients including cell type and clinical annotations were obtained from Maynard et al.^46^ which was analyzed using the Python package scanpy.^82^ The AXL signature score is defined as the average expression of genes in C1, C2, and C3, minus the average expression of a randomly sampled reference set of genes for each binned expression value by using the scanpy function “sc.tl.score_genes”. The Kaplan-Meier curves displaying the overall survival of LUAD and PAAD patients according to the gene expression of the AXL downstream signature were generated using the web server GEPIA2^83^ which uses TCGA/GTEx data.

#### Bacterial display library preparation

The sequences used to construct the ‘Mass Spectrometry’ library were derived from peptide sequences of key proteins detected by mass spectrometry. Suspected phosphorylation sites were identified in these proteins to create 11-residue peptide sequences, with 5 residues flanking a central tyrosine on each end. The central tyrosine was represented with a ‘TAT’ codon, and the surrounding peptide sequences were converted into DNA sequences that are codon-optimized for *E. coli* usage, while avoiding SfiI restriction sites. For peptides containing tyrosine residues surrounding the phosphoacceptor tyrosine site, we substituted those surrounding tyrosine residues for phenylalanine. All sequences were then flanked with 5′-GCTGGCCAGTCTGGCCAG-3′ on the 5′ side and 5′-GGAGGGCAGTCTGGGCAGTCTG-3′ on the 3′ side, as oligos. This oligo pool was generated using on-chip massively parallel synthesis (Twist Bioscience), and PCR amplified with Oligo-pool forward (5′-GCTGGCCAGTCTGGC-3′) and reverse (5′-CAGACTGCCCAGACTGC-3′) primers over 10 cycles. All PCR reactions were done with Q5 high-fidelity DNA polymerase (NEB), and PCR products were subsequently purified with DNA clean-up after each PCR and restriction. DNA clean-up and gel extractions were performed with QiAquick Gel Extraction Kit (Qiagen).

This library is constructed with the pBAD33 plasmid vector, with an eCPX displaying gene tagged with c-Myc tag. The eCPX scaffold was PCR amplified with eCPX forward (5′-GGAGGGCAGTCTGGGCAGTCTG-3′) and eCPX reverse (5′-GGCTGAAAATCTTCTCTCATCCGCC-3′) primers to create a scaffold. This PCR product underwent a second PCR with the same eCPX Reverse primer, but with the oligo-pool library as the forward primer. The final PCR product library contained two SfiI restriction sites.

Simultaneously, the pBAD33 plasmid containing SfiI restriction sites flanking HindII and PstI restriction sites was digested with SfiI at 50°C for 1 h to generate the backbone. The reaction was then subjected to additional HindII and PstI digestion at 37°C for 1 h to remove undesired insert byproducts and undigested vector from the SfiI digest. The backbone was purified by gel electrophoresis. The PCR product was digested with SfiI at 50°C for 1 h, followed by DNA clean-up to generate the insert.

The purified library insert was ligated into the digested pBAD33-eCPX backbone using T4 DNA ligase (NEB) for 1 h at 25°C. The ligation involved 50 ng of DNA, with a 1:3 molar ratio of backbone:insert. The ligation reaction was used to transform commercial MC1061F cells by electroporation and purified using the QIAprep Spin Miniprep Kit (Qiagen).

#### Preparation of E. Coli for peptide display of different peptide libraries

25 μL of electrocompetent E. coli SS320 (MC1061 F′) cells were transformed with 200 ng of library DNA, and the cells recovered with 1 mL of warm LB at 37°C for 1 h with shaking. These cells were resuspended in 250 mL of LB/chloramphenicol and incubated overnight at 37°C. 90 μL of the overnight culture was used to inoculate 5 mL of LB/chloramphenicol. This culture was grown at 37°C for 1–2 h until the cells reached an optical density of 0.5 at 600 nm, where 0.2% Arabinose was introduced for the induction of eCPX and peptide libraries. The culture was incubated for 4 h at 25°C. Afterward, small aliquots (75 μL) of the culture were pelleted via centrifuge at 4000 g for 15 min, and the pellets were resuspended with 50 μL of kinase screen buffer (50 mM Tris, 10 mM MgCl_2_, 150 mM NaCl, 2 mM Na_3_VO_4_ and 1 mM tris (2-carboxyethyl) phosphine).

#### Evaluation of AXL library phosphorylation by flow cytometry

AXL kinase was obtained commercially from Carna Bioscience, as GST-AXL (GST-Tagged) and BTN-AXL (Biotin Tagged). 2.7 μM of the kinase was subject to auto-phosphorylation with 5 mM of ATP and 10 mM MgCl_2_, for one hour at room temperature. 5 μL of the auto-phosphorylated kinase was added to 50 μL of resuspended cells displaying peptides on eCPX. The mixture was heated to 37°C for 2 min, and 0.5 μL of 100 mM ATP was added to the reaction mixture. To evaluate AXL kinase activity 10 μL aliquots were removed from the reaction mixture at 5-, 10-, 20-, 40-, and 80-min intervals. For the final assay, 30 min reaction time was used. The reaction mixtures were quenched with introduction of 25 mM EDTA to aliquots and cooling at 4°C. Subsequently, the cells were pelleted at (1500 g, 20 min), and washed with 20 μL PBS with 0.2% bovine serum albumin (BSA), before being pelleted as a precursor to antibody incubation. To evaluate the efficiency and kinetics of the phosphorylation reaction, the 10 μL pellets from 5-, 10-, 20-, 40-, and 80-min reaction intervals were labeled by resuspension with 1:25 dilution of the PY20-PerCP-eFluor 710 conjugate (eBioscience) and 9E10 Alexa Fluor 488 conjugate (Invitrogen) in PBS containing 0.2% BSA for 1 h on ice, in the dark. These antibodies revealed the level of phosphorylation and myc (control, reference epitope) expression respectively. The cells were subsequently pelleted, and washed again with PBS with 0.2% BSA, before being diluted in 500 μL of PBS with 0.2% BSA for subsequent analysis using flow cytometry (BD LSR-Fortessa Cell Analyzer). To achieve similar library kinases phosphorylation levels as described previously,^62^ an optimal concentration of kinase and reaction time was determined to achieve around 30% library phosphorylation (Figures S5G-S5I).

#### Magnetic beads pulldown

100 μL of cells phosphorylated by AXL kinase (both GST-AXL, BTN-AXL) for 30 min was quenched and washed as described in ‘Preliminary assays to evaluate AXL phosphorylation’. The pelleted cells were resuspended on ice with 100 μL of 0.2% BSA, 1 mg/mL (1:500 dilution) of biotinylated anti-phosphotyrosine 4G10 (Merck) antibody and incubated for 1 h. The cells were washed and resuspended with 0.1% BSA, 2 mM EDTA in PBS (Isolation buffer).

Following this, streptavidin beads (Dynabeads FlowComp Flexi Fit, Thermo Fisher) were rinsed with 1 mL of isolation buffer. 450 μL of isolation buffer were added to 50 μL of the antibody-labelled cells, and 75 μL of washed streptavidin beads were added. The mixture was rotated for 30 min at 4°C. Another 375 μL of isolation buffer was added to the beads-cell mixture before the beads were pulled down via a magnetic rack. The supernatant was removed, and the remaining beads were resuspended in 1 mL of isolation buffer and rotated for 30 min at 4°C. The beads were pulled down again with a magnetic rack and resuspended in 100 μL of water. These beads were boiled for 10 min at 100°C to lyse the cells. The mixture was centrifuged to pellet the beads, and the supernatant was extracted to obtain DNA for deep sequencing. In addition, 50 μL of cells from the PBS resuspension which were not labeled with antibodies were also boiled for 10 min at 100°C to obtain DNA that was input into the cells.

#### Deep sequencing and enrichment detection

20 μL of the lysate DNA containing the peptide library was PCR amplified with Q5 high-fidelity DNA polymerase (New England Biolabs), for 15 cycles in a 50 μL reaction, with Lib Prep 1 Forward (5′-TGACTGGAGTTCAGACGTGTGCTCTTCCGATCTNNNNNNNGCAGGTACTTCCGTAGC-3′) and Lib Prep 1 Reverse (5′-CACTCTTTCCCTACACGACGCTCTTCCGATCTNNNNNNNTTTTGTTGTAGTCACCAGACTG-3′). 0.33 μL of the PCR product was then used for a second PCR reaction to attach the TruSeq UDI (Illumina) indexes, via a 20 cycle 50 μL PCR reaction with Q5 high-fidelity DNA polymerase (NEB), using Lib Prep 2 Forward (5′-CAAGCAGAAGACGGCATACGAGATGTGACTGGAGTTCAGACGTG-3′) and Lib Prep 2 Reverse (5′-AATGATACGGCGACCACCGAGATCTACACACACTCTTTCCCTACACGAC-3′) oligos. The resulting PCR product was purified using SPRIselect beads (Beckman Coulter). The samples were then submitted to the for Miseq and Novaseq deep sequencing (Illumina). Two technical replicates were performed on cells from the same library transformation, and two biological replicates were performed on cells from different library transformations, done on different days.

#### Analysis and software

FLASH software was used to merge pair-end reads, and cutadapt was used to remove flanking sequences surrounding the variants of interest. To calculate enrichment scores of each peptide variant, the following equation was used.


$$Frequency_{peptide}=\frac{n_{peptide}}{n_{total}}$$

$$Enrichment_{peptide}=\frac{Frequency_{peptide\;pull\;down}}{Frequency_{peptide\;input}}$$

The number of reads of each peptide of each sample ($n_{peptide}$) was first normalised as a frequency value $Frequency_{peptide}$ to the total number of reads for every peptides encoded by the library ($n_{total}$). Then, $Frequency_{peptide}$ was compared between the pull down ($Frequency_{peptidepulldown}$) and input ($Frequency_{peptideinput}$), as a log 2-fold change ratio. To compare enrichment scores between replicates, the Z score of each peptide’s enrichment was calculated, using:

$$Z_{enrichment}=\frac{Enrichment_{peptide}-\mu_{Enrichment}}{\sigma_{Enrichment}}$$

Where $\sigma_{Enrichment}$ is the standard deviation of the library’s enrichment score of the replicate, and $\mu_{Enrichment}$ is the standard deviation of the library’s enrichment score.

#### Kinase activity assay

The assay reaction mixture consists of 50 mM Tris pH 7.5, 0.1 mM EGTA, 1 mM DTT, 10 mM MgCl_2_, 2 mM MnCl_2_ and 4 mM NaVO_3_. 100 nM of AXL and FAK2 (Carna Bioscience) were initially individually incubated with 50 μM of cold ATP separately for 30 min at 30°C for autophosphorylation. Then, the two kinases were chilled to 4°C and mixed to achieve a final concentration of 50 nM for each kinase. 0.1 μCi/μL of [γ33P]-ATP (Hartmann Analytic) was added to the reaction mixture, and the reaction was carried out at 30°C for 1 and 5 min, respectively. The reaction was quenched by adding SDS-PAGE buffer at the designated time points. The proteins were then separated by SDS-PAGE. Incorporated 33P signals were detected using an Amersham Typhoon 5 Biomolecular Imager (GE Healthcare Life Sciences) and quantified using ImageQuant TL-10.2.4 (Amersham Biosciences). All kinase assays were performed in triplicate.

#### Drug-tolerant persister cell assay

1,000 cells were seeded in a 96-well plate and then treated with the indicated treatments and cell lines, with four technical replicates per treatment. XAV-939 is a tankyrase inhibitor that is widely used as an indirect inhibitor of YAP activity.^65,69,70^ After 15 days, the treatment solutions were replaced with complete RPMI-1640 media for an additional 15 days to allow drug-tolerant persister cells to regrow. Cells were imaged every 4 h using an IncuCyte S3 (Essen Bioscience) at 10× magnification, with 4 images per well. Phase images were manually thresholded and then analyzed by IncuCyte S3 software (Essen Bioscience) to determine cell confluency. At day 30 of the DTP assay, PC9 DTP PAR and DTP AXL KO cells were harvested and re-seeded into 96-well plates, alongside freshly thawed PC9 PAR and AXL KO cells as controls. The day after, cells were treated with the indicated titration of E for 3 days before quantifying cell viability using a CellTiter-Glo assay.

### QUANTIFICATION AND STATISTICAL ANALYSIS

Unless otherwise indicated, statistical analyses were performed using Python. For comparisons between two groups, statistical significance was assessed using two-sided Mann–Whitney U tests or unpaired Student’s t-tests, as indicated in the figure legends or relevant Methods subsections. For phenotypic measurements of cell viability, apoptosis, and migration, E- and EA-treated conditions were compared across measured time points using t-tests. For the cell island phenotype, statistical significance was calculated using Ripley’s K function at a radius of 1 μm, followed by a *t* test comparing treatment conditions. For each phenotypic measurement, *n* = 3. Survival differences in Kaplan–Meier analyses were assessed using log rank tests. In Figures 5D, 5E, and 5H-5K, statistical significance was calculated using Mann-Whitney U rank tests, whereas a Student’s *t* test was used in J. In J–K, the error bars are defined by the standard error of the mean. In Figures 6I-6H, Correlations between patient-level signature scores were evaluated using Pearson correlation coefficients, with significance estimated by permutation testing using 10,000 label permutations. Error bars or shaded regions represent the standard error of the mean. Statistical significance is denoted as follows: *p < 0.05, **p < 0.01, ***p < 0.001, ****p < 0.0001; ns, not significant.

## Supplementary Material

1

Supplemental information can be found online at https://doi.org/10.1016/j.celrep.2026.117717.

## Figures and Tables

**Table:** KEY RESOURCES TABLE

REAGENT or RESOURCE	SOURCE	IDENTIFIER
Antibodies		
Rhodamine-conjugated β-tubulin	Bio-Rad	AbD22584
AXL-activating/primary antibody	R&D Systems	AF154; RRID: AB_354852
Biotinylated AXL antibody	R&D Systems	BAF154; RRID: AB_2062559
Primary AXL antibody	Cell Signaling Technology	8661; RRID: AB_11217435
Pan-tyrosine biotinylated antibody	R&D Systems	BAM1676; RRID: AB_356879
Primary antibody YAP S127-p	Cell Signaling Technology	4911; RRID: AB_331398
Primary antibody YAP	Santa Cruz	sc-101199; RRID: AB_1131430
Alexa Fluor 488 goat anti-rabbit IgG	Thermo Fisher	A-11008; RRID: AB_143165
PE goat anti-mouse IgG	Thermo Fisher	31861; RRID: AB_429715
PT-66	Millipore Sigma	P3300; RRID: AB_477335
PY20-PerCP-eFluor 710 conjugate	Thermo Fisher	46-5001-42; RRID: AB_2573761
9E10 Alexa Fluor 488 conjugate	Thermo Fisher	MA1-980-A488; RRID: AB_2609819
Biotinylated anti-phosphotyrosine 4G10	Merck Millipore	05-321
Bacterial and virus strains		
E. coli SS320 (MC1061 F′) cells	Invitrogen	C66303
Chemicals, peptides, and recombinant proteins		
Erlotinib	LC Laboratories	E-4997
CEP-40783	Selleck Chemicals	RXDX-106
XAV-939	Selleck Chemicals	XAV-939
Dasatinib	LC Laboratories	D-3307
Trametinib	MedChem Express	HY-10999
YOYO-3	Thermo Fisher Scientific	Y3606
Collagen I solution	Thermo Fisher Scientific	A1048301
TMT10plex	Thermo Fisher Scientific	90406
GST-AXL (GST-Tagged)	Carna Bioscience	N/A
BTN-AXL (Biotin Tagged)	Carna Bioscience	N/A
FAK2	Carna Bioscience	N/A
[γ33P]-ATP	Hartmann Analytic	HF-301
Streptavidin beads (Dynabeads FlowComp Flexi Fit)	Thermo Fisher	11061D
SPRIselect beads	Beckman Coulter	B23318
Critical commercial assays		
Luminex kit EGFR Y1068-p	Bio-Rad Bio-Plex	N/A
Luminex kit *p*-AKT S473-p	Bio-Rad Bio-Plex	N/A
BCA protein concentration assay	Pierce	A55866
High-Select Fe-NTA enrichment kit	Pierce	A32992
RNeasy Mini Kit	Qiagen	74104
QiAquick Gel Extraction Kit	Qiagen	28704
QIAprep Spin Miniprep Kit	Qiagen	27104
CellTiter-Glo assay	Promega	G7570
Deposited data		
Omics data of human LUAD patients	CPTAC LUAD study	Gillette et al.^45^
scRNA-seq of LUAD patients	Maynard et al.^46^	BioProject- PRJNA591860
Bulk RNA-seq of PC9 AXL Y-to-F lines	This study	SRA: PRJNA1012098 GEO: GSE333379
Phosphoproteomics data of PC9 AXL Y-to-F lines	This study	https://doi.org/10.6019/PXD079076
Experimental models: Cell lines		
PC9 cells	Sigma Aldrich	90071810-1VL
HEK293T cells	Sigma Aldrich	12022001-DNA-5UG
PC9 YAP KO cells	Passi Jänne’s laboratory	N/A
PC9 AXL KO cells	This paper	N/A
PC9 AXL Y-to-F mutant cell lines	This paper	N/A
Oligonucleotides		
eCPX forward (5′ - GGAGGGCAGTCTGGGCAGTCTG - 3′)	IDT	N/A
eCPX reverse (5′ - GGCTGAAAATCTTCTCTCATCCGCC - 3′)	IDT	N/A
PCR1 forward (5′- CACTCTTTCCCTACACGACGCTCTTCCGATCTCCGCGG	IDT	N/A
TTTTTTGTTGTAGTCACCAGACTG - 3′)		
PCR1 reverse (5′- TGACTGGAGTTCAGACGTGTGCTCTTCCGATCTTTATAACCGCAGGTACTTCCGTAGC - 3′)	IDT	N/A
PCR2 forward 01(5′ - CAAGCAGAAGACGGCATACGAGATCCGCGGTTGTGACTGGAGTTCAGACGTG - 3′)	IDT	N/A
PCR2 forward 02(5′ - CAAGCAGAAGACGGCATACGAGATTTATAACCGTGACTGGAGTTCAGACGTG - 3′)	IDT	N/A
PCR2 forward 03(5′ - CAAGCAGAAGACGGCATACGAGATGGACTTGGGTGACTGGAGTTCAGACGTG - 3′)	IDT	N/A
PCR2 forward 04(5′ - CAAGCAGAAGACGGCATACGAGATAAGTCCAAGTGACTGGAGTTCAGACGTG - 3′)	IDT	N/A
PCR2 forward 05(5′ - CAAGCAGAAGA CGGCATACGAGATATCCACTGGTGACTGGAGTTCAGACGTG - 3′)	IDT	N/A
PCR2 forward 06(5′ - CAAGCAGAAG ACGGCATACGAGATGCTTGTCAGT GACTGGAGTTCAGACGTG - 3′)	IDT	N/A
PCR2 forward 07(5′ - CAAGCAGAAGACGGCATACGAGATCAAGCTAGGTGACTGGAGTTCAGACGTG - 3′)	IDT	N/A
PCR2 forward 08(5′ - CAAGCAGAAGACGGCATACGAGATTGGATCGAGTGACTGGAGTTCAGACGTG - 3′)	IDT	N/A
PCR2 forward 09(5′ - CAAGCAGAAGACGGCATACGAGATAGTTCAGGGTGACTGGAGTTCAGACGTG - 3′)	IDT	N/A
PCR2 forward 10(5′ - CAAGCAGAAGACGGCATACGAGATGACCTGAAGTGACTGGAGTTCAGACGTG - 3′)	IDT	N/A
PCR2 forward 11(5′ - CAAGCAGAAGACGGCATACGAGATTCTCTACTGTGACTGGAGTTCAGACGTG - 3′)	IDT	N/A
PCR2 forward 12(5′ - CAAGCAGAAGACGGCATACGAGATCTCTCGTCGTGACTGGAGTTCAGACGTG - 3′)	IDT	N/A
PCR2 forward 13(5′ - CAAGCAGAAGACGGCATACGAGATCCAAGTCTGTGACTGGAGTTCAGACGTG - 3′)	IDT	N/A
PCR2 forward 14(5′ - CAAGCAGAAGACGGCATACGAGATTTGGACTCGTGACTGGAGTTCAGACGTG - 3′)	IDT	N/A
PCR2 forward 15(5′ - CAAGCAGAAGACGGCATACGAGATGGCTTAAGGTGACTGGAGTTCAGACGTG - 3′)	IDT	N/A
PCR2 forward 16(5′ - CAAGCAGAAGACGGCATACGAGATAATCCGGAGTGACTGGAGTTCAGACGTG - 3′)	IDT	N/A
PCR2 forward 17(5′ - CAAGCAGAAGACGGCATACGAGATTAATACAGGTGACTGGAGTTCAGACGTG - 3′)	IDT	N/A
PCR2 forward 18(5′ - CAAGCAGAAGACGGCATACGAGATCGGCGTGAGTGACTGGAGTTCAGACGTG - 3′)	IDT	N/A
PCR2 forward 19(5′ - CAAGCAGAAGACGGCATACGAGATATGTAAGTGTGACTGGAGTTCAGACGTG - 3′)	IDT	N/A
PCR2 forward 20(5′ - CAAGCAGAAGACGGCATACGAGATGCACGGACGTGACTGGAGTTCAGACGTG - 3′)	IDT	N/A
PCR2 forward 21(5′ - CAAGCAGAAGACGGCATACGAGATGGTACCTTGTGACTGGAGTTCAGACGTG - 3′)	IDT	N/A
PCR2 forward 22(5′ - CAAGCAGAAGACGGCATACGAGATAACGTTCCGTGACTGGAGTTCAGACGTG - 3′)	IDT	N/A
PCR2 forward 23(5′ - CAAGCAGAAGACGGCATACGAGATGCAGAATTGTGACTGGAGTTCAGACGTG - 3′)	IDT	N/A
PCR2 forward 24(5′ - CAAGCAGAAGACGGCATACGAGATATGAGGCCGTGACTGGAGTTCAGACGTG - 3′)	IDT	N/A
PCR2 forward 25(5′ - CAAGCAGAAGACGGCATACGAGATACTAAGATGTGACTGGAGTTCAGACGTG - 3′)	IDT	N/A
PCR2 forward 26(5′ - CAAGCAGAAGACGGCATACGAGATGTCGGAGCGTGACTGGAGTTCAGACGTG - 3′)	IDT	N/A
PCR2 forward 27(5′ - CAAGCAGAAGACGGCATACGAGATCTTGGTATGTGACTGGAGTTCAGACGTG - 3′)	IDT	N/A
PCR2 forward 28(5′ - CAAGCAGAAGACGGCATACGAGATTCCAACGCGTGACTGGAGTTCAGACGTG - 3′)	IDT	N/A
PCR2 forward 29(5′ - CAAGCAGAAGACGGCATACGAGATCCGTGAAGGTGACTGGAGTTCAGACGTG - 3′)	IDT	N/A
PCR2 forward 30(5′ - CAAGCAGAAGACGGCATACGAGATTTACAGGAGTGACTGGAGTTCAGACGTG - 3′)	IDT	N/A
PCR2 forward 31(5′ - CAAGCAGAAGACGGCATACGAGATGGCATTCTGTGACTGGAGTTCAGACGTG - 3′)	IDT	N/A
PCR2 forward 32(5′ - CAAGCAGAAGACGGCATACGAGATAATGCCTCGTGACTGGAGTTCAGACGTG - 3′)	IDT	N/A
Recombinant DNA		
AXL-IRES-Puro vector	Addgene	65627
Lentiviral vector with puromycin resistance marker	Addgene	17448
VSV-G envelope vector	Addgene	12259
Packaging vector	Addgene	12260
pBAD33 plasmid vector	Addgene	36267
Software and algorithms		
IncuCyte S3 software	Essen Bioscience	V2022A
ImageJ	ImageJ.org	1.54i
astropy	astropy.org	7.2
Proteome Discoverer 2.5	Thermo Fisher Scientific	2.5
Mascot 2.4	MatrixScience	RRID:SCR_014322
scikit-learn	Scikit-learn.org	1.7
gseapy	https://github.com/zqfang/GSEApy	V1.1.10
scanpy	https://github.com/scverse/scanpy	V1.12.0
GEPIA2	http://gepia2.cancer-pku.cn/	N/A
FLASH	https://github.com/ebiggers/flash	N/A
cutadapt	https://github.com/marcelm/cutadapt	N/A
ImageQuant TL-10.2.4	Amersham Biosciences	N/A
AXLomics code	This paper	https://doi.org/10.5281/zenodo.20274687
Other		
IncuCyte S3	Essen Bioscience	N/A
BD FACSAria cell sorter	BD Biosciences	N/A
Q Exactive HF-X mass spectrometer	Thermo Fisher Scientific	N/A
Agilent 1260 LC system	Agilent	N/A
Q Exactive Plus mass spectrometer	Thermo	N/A
Miseq and Novaseq	Illumina	N/A
BD LSR-Fortessa Cell Analyzer	BD Biosciences	N/A
Amersham Typhoon 5 Biomolecular Imager	GE Healthcare Life Sciences	N/A

## Highlights

- AXL Y-to-F mutants and multivariate modeling link specific signaling to phenotypes
- Two phosphotyrosine clusters drive PC9 cell viability and migration
- AXL engages a FAK1/SFK-Abl1/YAP axis to drive the emergence of drug-tolerant persister cells
- Downstream AXL signaling is active in EGFRm LUAD patients who do not respond to therapy
