# Supplementary material for: AXL phosphosite mapping links FAK1 and YAP1 signaling to erlotinib resistance in EGFR-mutant lung cancer
Source: Cell Rep. Author manuscript; Available in PMC 2026 Aug 17. (PMC13480988; doi:10.1016/j.celrep.2026.117717)
Supplement: Supplementary file 1 [file NIHMS2199978-supplement-1.pdf]

Cell Reports, Volume *45*

Supplemental information

AXL phosphosite mapping links FAK1 and YAP1 signaling to erlotinib resistance in EGFR-mutant lung cancer

Marc Creixell, Scott D. Taylor, Jacqueline Gerritsen, Mingxuan Jiang, Song Yi Bae, Teresa Augustin, Michelle Loui, Carmen Boixo, Saki Minamino, Victoria Gong, Pau Creixell, Forest M. White, and Aaron S. Meyer

SUPPLEMENTARY INFORMATION

Pilot experiment to select AXL intracellular tyrosines to generate stable Y-to-F cell lines

With this goal in mind, we selected Y-to-F mutants that conferred different degrees of survival capacity to erlotinib. Prior to establishing stable, lentivirally-transduced Y-to-F AXL mutant PC9 cell lines, we performed a pilot experiment to identify AXL intracellular tyrosines based on a selection-based FACS assay. In this assay, HCC827 AXL KO cells were transiently transfected with an AXL (Y-to-F)-IRES-GFP plasmid and treated with erlotinib (E) to quantify the enrichment of AXL/GFP+ HCC827 cells in response to treatment, normalized to HCC827 AXL kinase dead (K567R, KD) cells. Y726F and Y750F led to >50% increase in the number of AXL/GFP+ cells induced by E whereas Y634F, Y643F, or Y821F abolished this AXL-mediated response, effectively behaving like cells expressing AXL KD (**Fig. S1A/B**). We selected Y-to-F mutants that conferred varying degrees of erlotinib resistance. This, along with existing knowledge of these sites in the literature, led us to choose positions 634, 643, 698, 726, 750, and 821 to generate stable AXL Y-to-F mutant cell lines in PC9.

PC9 cells are ideally suited for this study for two main reasons: First, these cells express AXL at baseline but lack robust receptor activation. This provides a signaling landscape where AXL downstream pathways are not triggered unless the receptor is exogenously activated. Second, PC9 is readily sensitive to E, which enabled us to induce oncogenic phenotypic responses through AXL activation during EGFR inhibition. To generate stable PC9 AXL mutant cell lines, we knocked out AXL in PC9 parental (PAR) cells using CRISPR/Cas9 and then lentivirally transduced either AXL wild-type (WT), AXL KD, or a Y-to-F AXL mutant insert (Y634F, Y643F, Y698F, Y726F, Y750F, or Y821F) (**Fig. 1A**). We confirmed AXL total abundance, cell-surface abundance, and activation in each cell line (**Fig. S1C/D**). We observed that ~25% of the transduced PC9 cells express AXL, yet the total AXL expressed and measured by western blot is at least equal to PC9 PAR. This suggests that each transduced AXL-expressing cell possesses on average 4-fold more AXL compared with PAR cells (**Fig. S1C**). While this is a caveat of our methodology, the higher AXL expression of the PC9 Y-to-F mutant cells is still within the range of the reported AXL expression in other EGFRm lung cancer cell lines such as H2279 and H4006[1]. Moreover, because the proportion of AXL KO cells in each of the PC9 Y-to-F cell line is comparable, cells lacking AXL will provide a consistent background signal, allowing for clear detection of phosphorylation changes driven by the PC9 AXL Y-to-F mutants.

AXL activation is challenging to faithfully manipulate given its dependence on phosphatidylserine (PS) present in apoptotic bodies and the spatially heterogeneous presentation of its ligand Gas6[2] . We and others have characterized why, although Gas6 binds AXL with high affinity, this is insufficient for physiological activation of the receptor without PS[1–3]. While AXL can become activated without exogenous addition of PS liposomes, this typically happens with a high level of AXL expression wherein cells develop constitutive activation of the receptor and are consequently poorly responsive[1–3]. Therefore, we used the AXL-activating antibody AF154 (A) to ensure that the receptor is maximally activated in a transient fashion so that we can observe the subsequent signaling response.

We did not detect AXL phosphorylation in AXL KO and AXL KD cells, whereas WT and the Y-to-F mutants showed different levels of kinase activity relative to each other. Notably, WT and the Y-to-F mutants displayed lower activation levels than PC9 PAR cells, which indicates that despite the increased AXL expression in PC9-transduced cells, the net amount of activated receptor is still higher in PAR cells (**Fig. S1D**). As expected, we observed complete inhibition of p-EGFR upon E treatment (**Fig. S1E**).

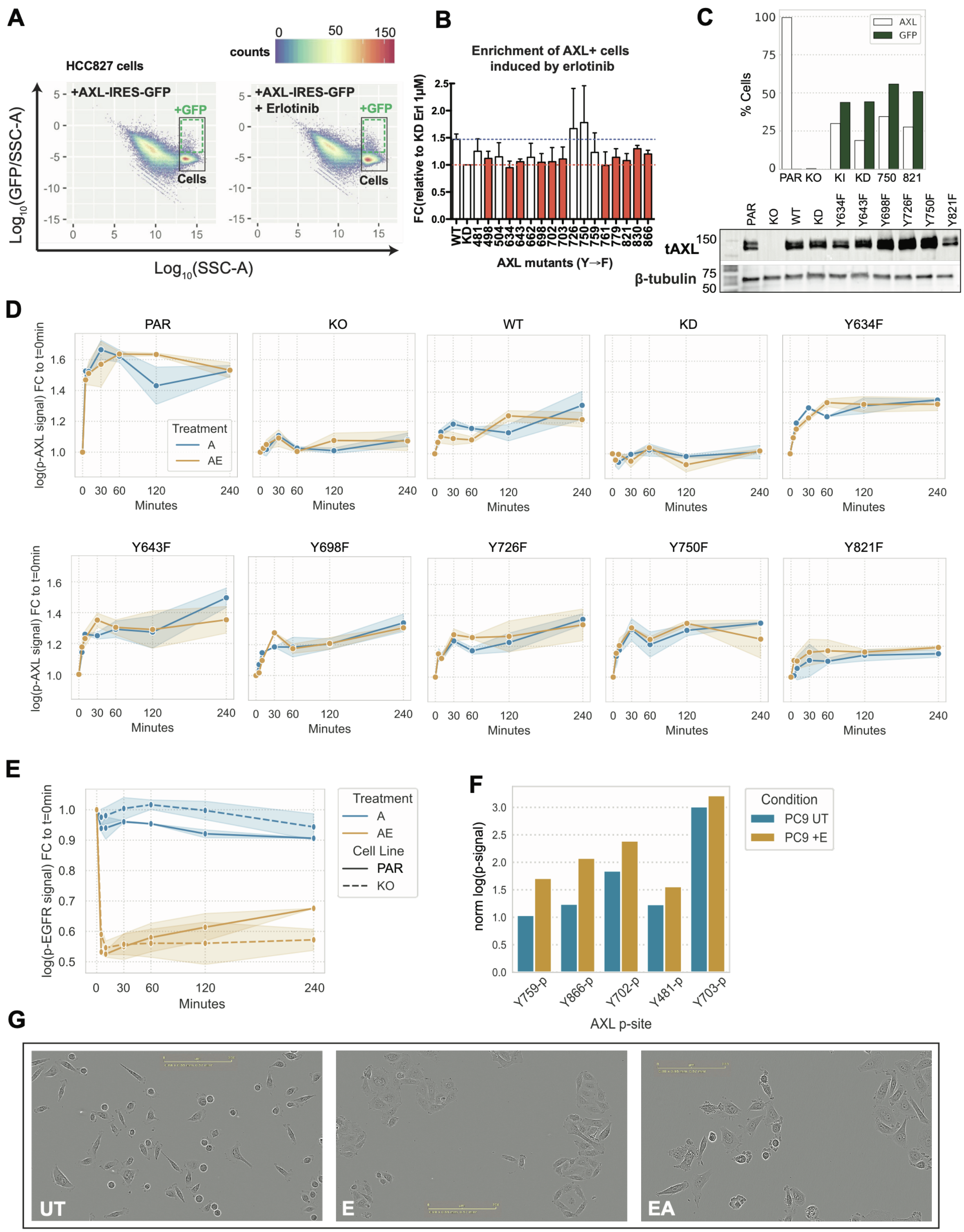

Figure S1. AXL expression and activation across PC9 AXL Y-to-F mutant cell lines. (A) To decide what AXL intracellular tyrosines to mutate and establish stable AXL mutant Y-to-F cell

lines we performed a pilot experiment in HCC827 cells. We transiently transfected HCC827 cells with an AXL (Y-to-F)-IRES-GFP cassette and then, after two days of E treatment, we quantified the amount of E-induced enrichment in AXL-expressing cells for each of the Y-to-F mutants. The axes in Fig. S1A represent GFP positivity which is a readout for AXL expression (y axis) and log-transformed side scatter (x axis, SSC-A). The green box is a gate that identifies the population of HCC827 cells expressing GFP/AXL. (B) Fold change of AXL+ cells in erlotinib versus untreated in each indicated Y-to-F mutant, normalized to the number of AXL KD+ cells treated with erlotinib. Red bars indicate AXL mutants that failed to induce the enrichment of GFP/AXL+ cells in response to erlotinib, whereas white bars correspond to AXL mutants that increased the GFP/AXL+ fraction of cells in the presence of treatment. We observed that Y726F and Y750F led to >50% increase in the number of AXL/GFP+ cells induced by E whereas Y634F, Y643F, or Y821F abolished this AXL-mediated response, effectively behaving like cells expressing AXL KD (B). (C) Cell surface and total protein AXL expression in the indicated PC9 cell lines measured by either FACS (top) or western blotting (bottom). (D-E) Elisa-based Luminex was used to quantify protein and phosphosite abundance. In both figures, PC9 cells were treated with 1uM E for 4h and EA (1μM E and 300ng/mL A) for 10 minutes or only with 300 ng/mL A for 10 minutes. (D) AXL phosphorylation signal in PC9 AXL mutant cell lines in response to A alone or in combination with E. Error bars show the standard error of the mean. (E) EGFR inhibition in response to E. (F) MS-measured phosphorylation abundances of AXL phosphosites in PC9 PAR cell untreated (UT) or treated with 1μM E for 4h. (G) Representative images of the E-induced island effect in PC9 PAR cells after 48h of treatment. In D–F the error regions correspond to the standard error of the mean. Scale bars represent 200 μm. Related to **Figure 1**.

Using DDMC to cluster peptides by sequence similarity and phosphorylation abundance

We next applied Dual Data-Motif Clustering (DDMC), a computational approach we previously developed to enhance signaling network modeling[4] . While phosphoproteomic measurements provide data on both phosphorylation abundance and peptide sequence, these are typically analyzed in isolation. DDMC integrates these data types using a mixture model that probabilistically and simultaneously analyzes peptide phosphorylation variation and sequence motifs. Through an iterative expectation-maximization algorithm, the method optimizes cluster assignments based on the average features of member sequences and their abundance profiles.

We utilized DDMC to categorize 491 mass spectrometry-measured peptides (**Fig. 2A**) into distinct signaling clusters sharing similar features. A tunable weight parameter allows for the prioritization of either information source during cluster fitting; setting a weight of 0 reduces DDMC to a Gaussian Mixture Model (GMM) based solely on phosphorylation signal, whereas a high weight prioritizes peptide sequence (**Fig. 2B**). To link these signaling clusters to cellular responses, we employed Partial Least Squares Regression (PLSR), allowing DDMC to infer the upstream kinases regulating each cluster (**Fig. 2B**). We optimized hyperparameters—specifically cluster number, sequence weight, and PLSR components—by evaluating the model's ability to predict phenotypes. After assessing the interpretability of the top 5% of performing models, we selected a final model consisting of five clusters, a sequence weight of 2, and two PLSR components (**Fig. S2A–D**).

Pearson correlations between DDMC clusters, AXL phosphosites, and phenotypes

To associate AXL-mediated signaling clusters with phenotypes, we first correlated DDMC cluster centers with AXL phosphosites and phenotypic responses (**Fig. 2C**). Cluster C3 correlated most strongly with all AXL tyrosine phosphorylation, particularly Y866-p and Y821-p. These sites also correlated with C1 and C2, though to a lesser extent (**Fig. S2K**). C1, C2, and C3 correlated positively with cell viability and migration and negatively with apoptosis and the island effect, while C4 correlated modestly with apoptosis (**Fig. S2L**).

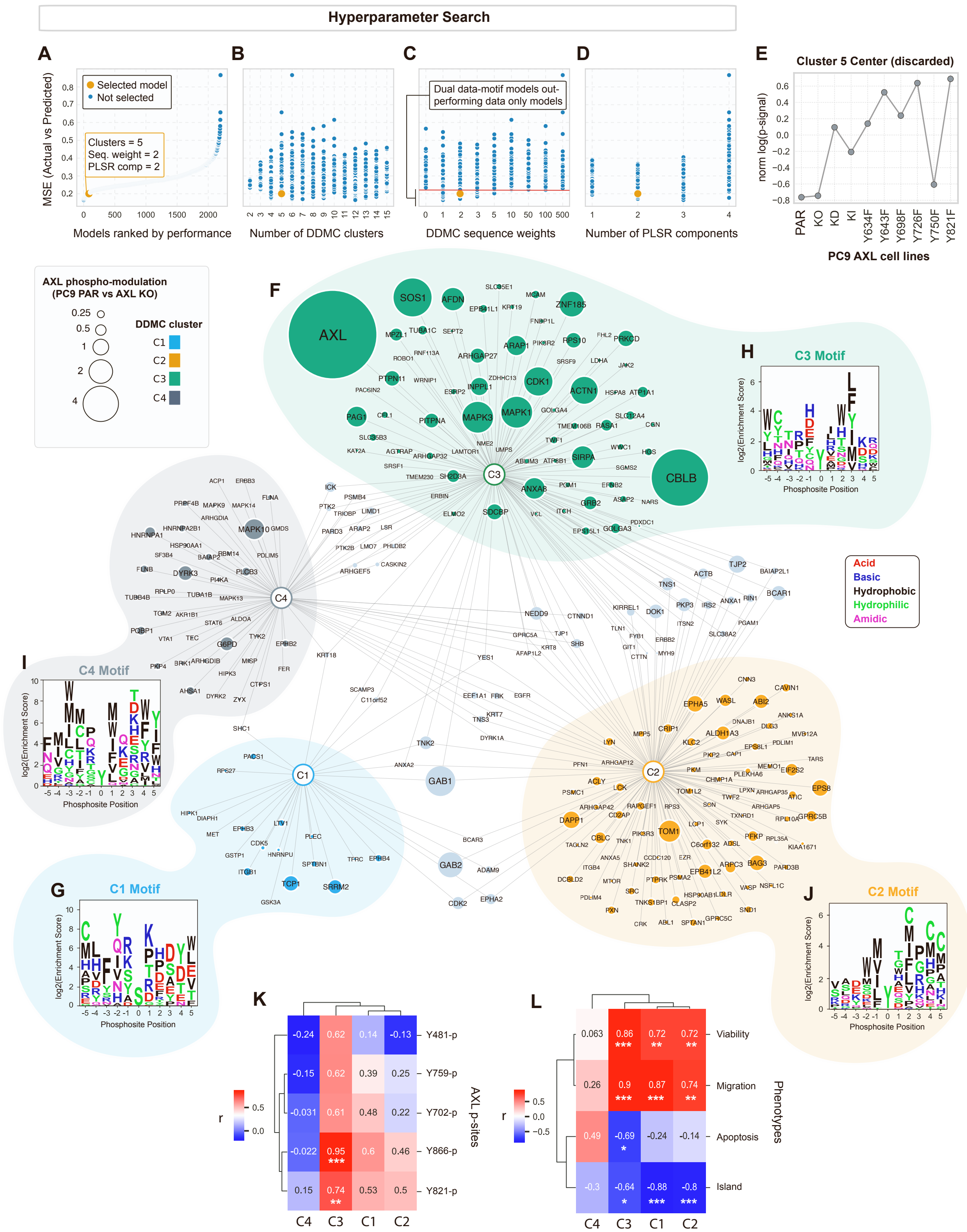

Figure S2. (A–D) Hyperparameter search based on the ability of DDMC centers to predict AXL-mediated phenotypes using PLSR. Mean-Squared Error (MSE) of actual versus predicted phenotypic measurements grouped by model performance (A), number of DDMC clusters (B),

DDMC sequence weights (C), and number of PLSR components (D). Note that even though some models were slightly more predictive, we selected the model using 5 clusters, a sequence weight of 2, and 2 PLSR components due to its interpretability. (E) DDMC Cluster 5 center which was discarded due to biologically inexplicable signaling behavior. (F) STRING protein network map of the top 50 proteins grouped by DDMC cluster and showing highest absolute differential of phospho-modulation in PC9 PAR cells compared with AXL KO cells. (G–J) Logo plots illustrating the PSSMs of each DDMC cluster. (K-L) Pearson correlations between DDMC clusters and AXL phosphosites (K) and DDMC clusters and AXL-mediated phenotypes (L). Related to **Figure 2**.

DDMC clustering and phenotype associations are independent of PC9 AXL expression

As seen in **Fig. S1C**, ectopic AXL expression varies across PC9 AXL Y-to-F mutant cell lines. This raises the question whether the identified signaling architecture, and hence subsequent phenotypic associations are influenced by this technical bias. To answer this question, we performed spearman correlation, orthogonal projection, and phenotype predictions (**Fig. S3**).

Spearman Correlation: AXL abundance vs DDMC centers

To directly test whether DDMC clusters reflect AXL dosage differences, we computed the Spearman rank correlation between normalized AXL protein abundance and each of the 5 DDMC cluster center profiles.

Importantly, for this analysis we excluded the AXL KO line from both the abundance vector and the cluster center profiles. We did so because the reviewer's concern pertains to variable ectopic AXL expression across the Y-to-F mutants. The KO represents a qualitatively different perturbation since it is a complete genetic ablation of the receptor, not a point on a dosage continuum. Including it would trivially anchor the correlation (near-zero abundance, strongly altered signaling) and confound the dosage-specific question.

The 95% CIs are wide because $n = 9$, and we observe that the point estimate for Cluster 1 ($r = +0.42$) cannot be statistically distinguished from zero. Moreover, the relevant question is whether we can rule out a strong correlation. None of the five clusters shows any such signal. Four clusters have point estimates $|r| < 0.30$, and Cluster 5 (the largest, at 183 peptides) shows a weak negative association, arguing against a uniform dosage effect across signaling programs. If AXL dosage were the dominant driver of a cluster's signaling program, dose would explain the majority of that cluster's cross-line variance, implying $r > 0.8$. With $n = 9$, a Spearman correlation test has approximately 77% power to detect $r = 0.8$ and 95% power to detect $r = 0.9$ at $\alpha = 0.05$ (two-tailed). That we observe no significant correlation across any of the 5 clusters, despite adequate power to detect a dominant effect, is therefore informative evidence against the dosage hypothesis. **Fig. S3B** shows labelled scatter plots for all 5 clusters.

Orthogonal Projection: Cluster Structure After Removing AXL Dosage Direction

In addition to the correlation analysis, we applied a more stringent analytical control: we completely removed the AXL abundance direction from the phosphoproteomic data matrix before re-clustering. Specifically, for every phosphopeptide profile x_i (a vector of length 10, one value per cell line), we computed its residual after orthogonal projection onto the unit vector of AXL abundance:

$$x_{i_{resid}} = x_i - \hat{u}_{AXL}(\hat{u}_{AXL} \cdot x_i)$$

where $\hat{u}\_AXL$ is the L2-normalised AXL abundance vector across all 10 cell lines. This operation removes, for every peptide, the component of its cell-line variation that is perfectly collinear with AXL protein level. As a sanity check, the maximum absolute inner product of any residual vector with $\hat{u}\_AXL$ was 2.22×10^{-16} (machine precision), confirming complete orthogonalisation.

We then re-ran DDMC on the residualised matrix using identical hyperparameters (5 clusters, SeqWeight = 2, PAM250 distance, random_state = 5).

Fig. S3C shows overlaid line plots of original (blue) vs. AXL-regressed (orange) centers for all 5 clusters, where each line depicts the mean phosphoproteomic signal of that cluster's peptides across the 10 cell lines. If a cluster's cross-line pattern were driven by AXL dosage ordering, removing the dosage direction would eliminate or distort the pattern — the two curves would diverge. Conversely, if the cluster program is independent of AXL dosage, the pattern persists after projection and the curves overlap. The near-perfect overlap of original and AXL-regressed centers therefore constitutes direct evidence that the cross-line phosphoproteomic programs are not shaped by AXL abundance variation; what drives the cluster patterns is a multivariate combination of phosphosite changes that are orthogonal to the AXL dosage axis.
To quantify this, we report the Pearson correlation between matched original and AXL-regressed center profiles:

Original → Residual: C1→C1, C2→C5, C3→C3, C4→C4, C5→C2

Phenotype Prediction: DDMC centers vs AXL Dosage

Finally, we compared the ability of three PLSR models to predict cellular phenotypes using leave-one-out (LOO) cross-validated Q^2 as the performance metric (**Fig. S3D**). LOO was chosen because n = 10 makes k-fold cross-validation unstable; LOO is the least biased estimator for such small samples. We assessed four phenotypes: Cell Viability, Apoptosis, Migration, and Island morphology, all measured under Erlotinib + AF154 (A/E) treatment.

The three models were:

1. Model A — DDMC cluster centers (5 features, 3 PLSR components (C)): This is the model used in the paper. The number of components (C=3) was selected by maximizing mean LOO Q^2 across C=1 to 5: C=1: 0.275, C=2: 0.390, C=3: 0.433, C=4: 0.311, C=5: −0.058.

2. Model B — AXL protein abundance only (1 feature, 1 PLSR component): With a single predictor variable, PLSR is constrained to exactly 1 component (additional components cannot be extracted from a rank-1 matrix); this is enforced by the algorithm and is equivalent to ordinary least squares regression. The difference in component count between Model A (3) and Model B (1) therefore reflects the difference in information content between the two feature sets, not an arbitrary modelling choice.

3. Model C — Cluster centers + AXL abundance (6 features, 3 PLSR components): Tests whether AXL dosage adds predictive information beyond the clusters.

Model A (DDMC cluster centers) yields positive LOO Q^2 for all four phenotypes, with particularly strong performance for Migration (Q^2 = 0.814). Model B (AXL dosage alone) is a negative predictor for every phenotype — its Q^2 is consistently below zero, meaning it predicts phenotypes worse than a naïve intercept-only model. This conclusively demonstrates that AXL protein abundance carries no useful predictive signal for the phenotypes under study; the predictive power of the DDMC clusters cannot be attributed to dosage effects.

Notably, Model C (clusters + AXL) does not improve over Model A for Viability and Apoptosis, and degrades for Apoptosis. This indicates that adding AXL abundance on top of the cluster centers introduces noise rather than signal — further evidence that the clusters capture information orthogonal to and independent of receptor dosage. Taken together, these three analyses provide multiple independent lines of evidence that DDMC clusters are not driven by AXL receptor dosage: (1) AXL protein abundance is uncorrelated with all 5 DDMC cluster center profiles across AXL-expressing lines (Spearman $|r| < 0.42$, all $p > 0.26$). (2) Analytical removal of the entire AXL dosage dimension from the phosphoproteomic matrix leaves the cluster center profiles — the programs used for all downstream analyses — robustly preserved: 4/5 clusters have center Pearson $r \geq 0.93$ with their original counterparts. (3) AXL abundance alone is a poor predictor of cellular phenotypes (mean LOO $Q^2 = -0.67$), DDMC cluster centers are substantially predictive (mean LOO $Q^2 = +0.43$). (4) Adding AXL abundance to the cluster model does not improve predictions, confirming that receptor dosage carries no independent information beyond what the clusters already encode. We conclude that the DDMC clusters reflect distinct, AXL dosage-independent phosphoproteomic signaling programs.

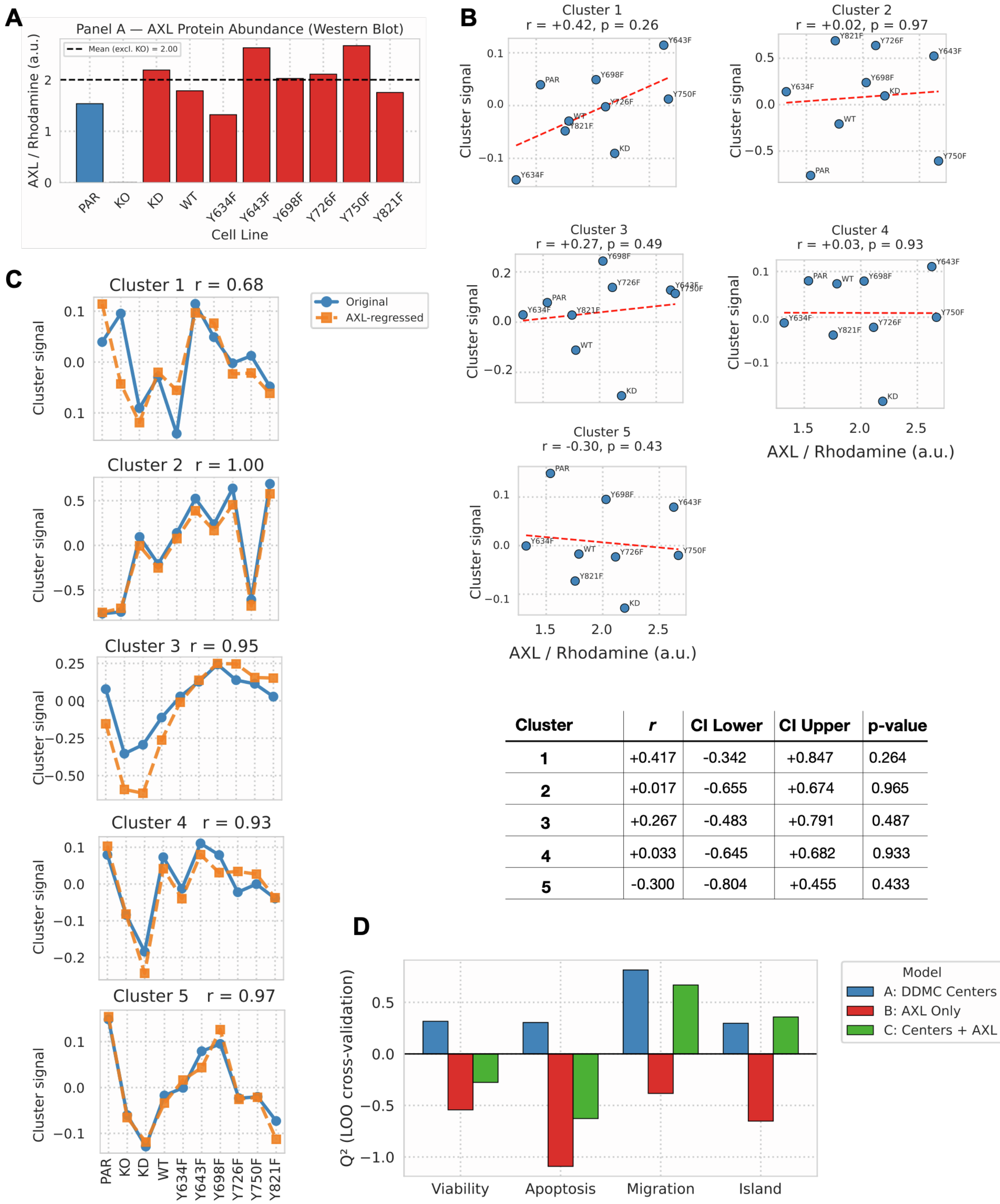

Cluster	*r*	CI Lower	CI Upper	p-value
1	+0.417	-0.342	+0.847	0.264
2	+0.017	-0.655	+0.674	0.965
3	+0.267	-0.483	+0.791	0.487
4	+0.033	-0.645	+0.682	0.933
5	-0.300	-0.804	+0.455	0.433

Figure S3. DDMC clusters reflect phosphoproteomic signaling programs independent of ectopic AXL expression. (A) AXL protein abundance across all 10 cell lines quantified by

western blot and normalized to Rhodamine total-protein stain as a loading control. KO (AXL-knockout) is shown in grey; all AXL-expressing lines are shown in red. Dashed line indicates the mean normalized AXL signal across AXL-expressing lines (excluding KO). (B) Spearman rank correlation between normalized AXL protein abundance and each of the five DDMC cluster center profiles across the nine AXL-expressing lines**.** Each scatter plot shows one cluster; the dashed red line is the OLS regression fit. Cell lines are individually labelled. Inset table reports Spearman r, 95% confidence intervals (Fisher z-transform approximation), and two-tailed p-values for all five clusters. None of the five clusters shows a significant correlation with AXL abundance (all $p > 0.26$). For context, a test at $n = 9$ has ~77% power to detect a dominant dosage effect ($r \geq 0.8$) at $\alpha = 0.05$ (two-tailed), indicating that the absence of significance constitutes meaningful negative evidence against such an effect. (C) Overlay of original DDMC cluster center profiles (blue) and cluster centers re-estimated after orthogonal projection of AXL abundance out of every phosphopeptide profile (orange, "AXL-regressed"). Each line shows the mean phosphoproteomic signal for that cluster's peptides across all 10 cell lines. Overlapping curves indicate that the cross-line phosphoproteomic pattern defining each cluster is orthogonal to — and therefore not driven by — AXL receptor abundance variation. (D) Leave-one-out (LOO) cross-validated Q^2 for three PLSR models predicting four cellular phenotypes (Cell Viability, Apoptosis, Migration, and Island morphology) measured under EA treatment. Model A: DDMC cluster centers (5 features, 3 PLSR components, selected by maximizing mean LOO Q^2); Model B: normalized AXL protein abundance only (1 feature, 1 PLSR component —the mathematical maximum for a single predictor, equivalent to ordinary least squares); Model C: cluster centers combined with AXL abundance (6 features, 3 PLSR components). Model A yields positive Q^2 for all four phenotypes (mean $Q^2 = +0.43$), whereas Model B performs worse than a naïve intercept-only predictor for all four phenotypes (mean $Q^2 = -0.67$), demonstrating that AXL abundance carries no independent predictive signal for the phenotypes of interest. Adding AXL abundance to the cluster model (Model C) does not improve predictions, further confirming that the dosage axis is orthogonal to the information encoded in the DDMC clusters. Related to **Figure 1** and **2**.

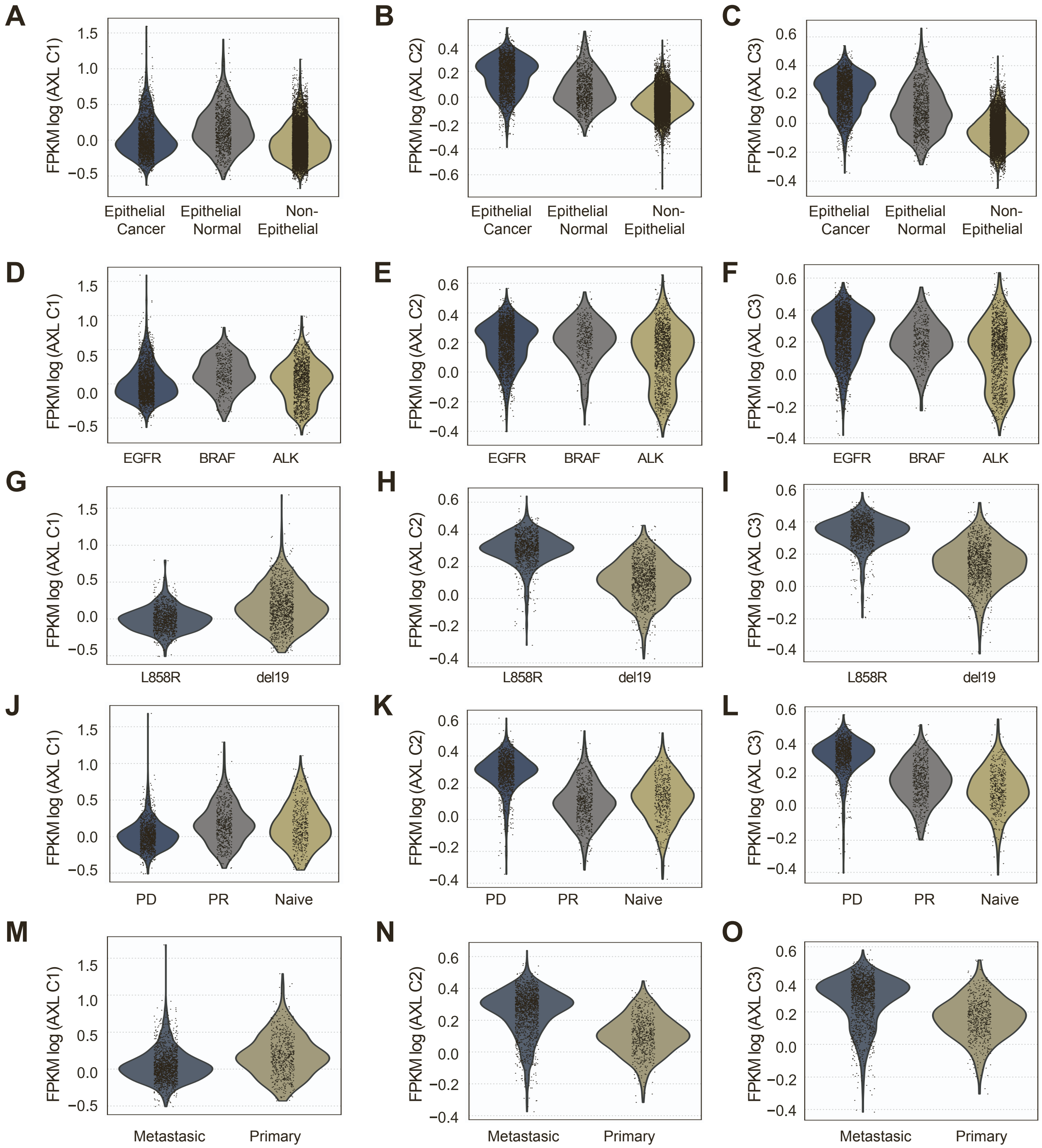

Figure S4 (A–O) Normalized gene expression of C1, C2, C3 in (A–C) epithelial cancer cells, epithelial normal cells, or non-epithelial; based on (D–F) gene driver, (G–I) EGFR mutation, (J–L) tumor response, and (M–O) metastasis status. Related to **Figure 3**.

Selection of AXL-BTN for in vitro kinase specificity screen

While screens using GST- and biotin (BTN)-tagged AXL yielded highly correlated specificities (Pearson's r = 0.96; **Fig. S4E**), we prioritized the AXL-BTN results to avoid tag-mediated cysteine enrichment observed with GST (**Fig. 5B/C and S4F/G**).

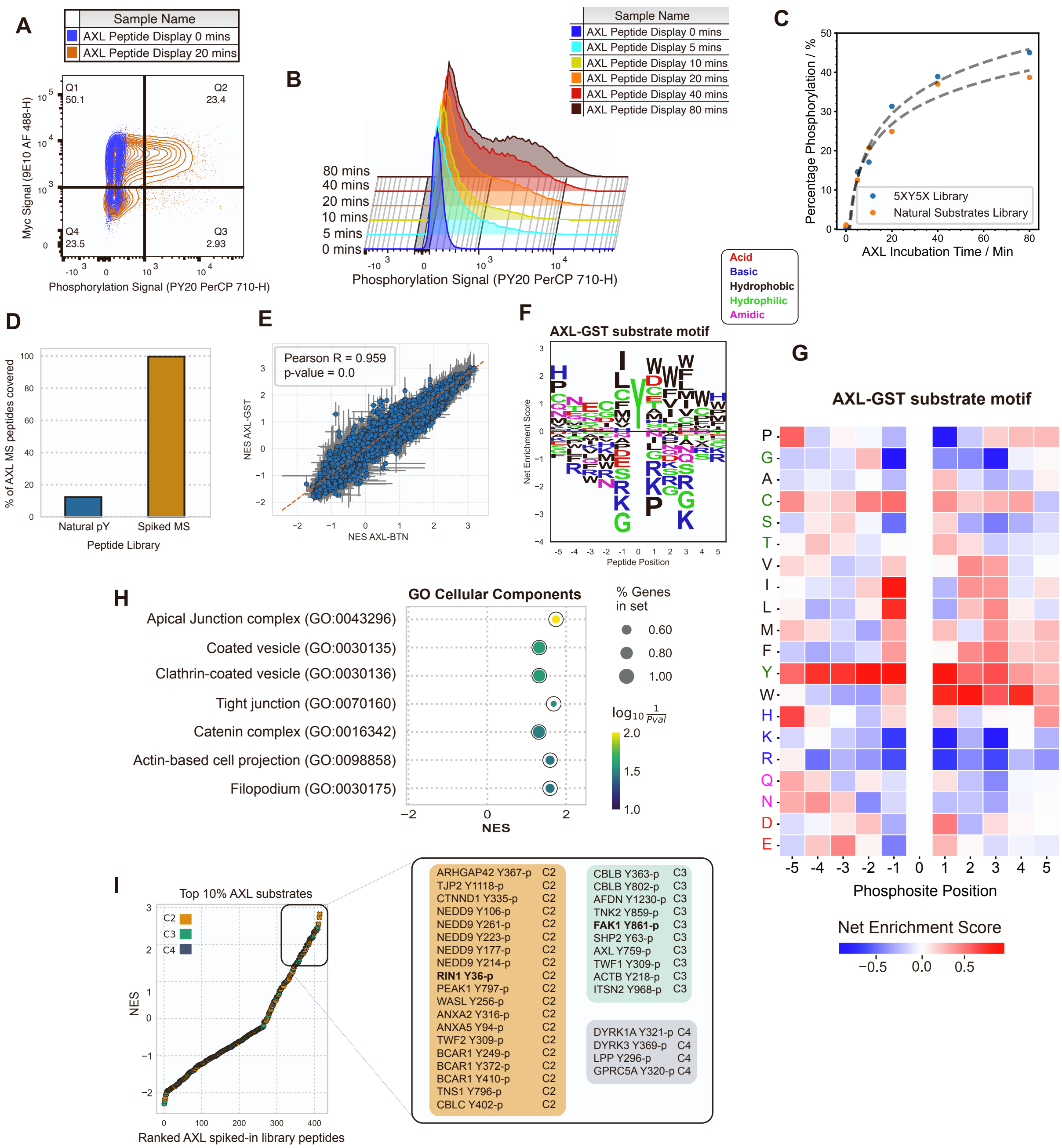

Figure S5. (A) Scatter plot of myc and phosphorylation signal of peptide display library in samples incubated during 0 or 20 minutes. (B) Histogram showing phosphorylation levels of peptide display library during incubations of 0, 5, 10, 20, 40, or 80 minutes. (C) Phosphorylation percentage as a function of time using two different peptide libraries (see methods for details). (D) Percentage of peptides covered by either the pTyr-Var library or after spiking with AXL-regulated peptides identified in phosphoproteomic analysis. (E) Pearson correlation of NES between AXL-GST and AXL-BTN. (F–G) AXL PSSM motif illustrated by either a logo plot (F) or a heatmap (G). (H) Ranked GSEA using the GO Cellular Components gene set. (I) Ranked AXL substrates by NES and colored by cluster membership, alongside a selected list of phosphosites per cluster. Related to **Figure 5**.

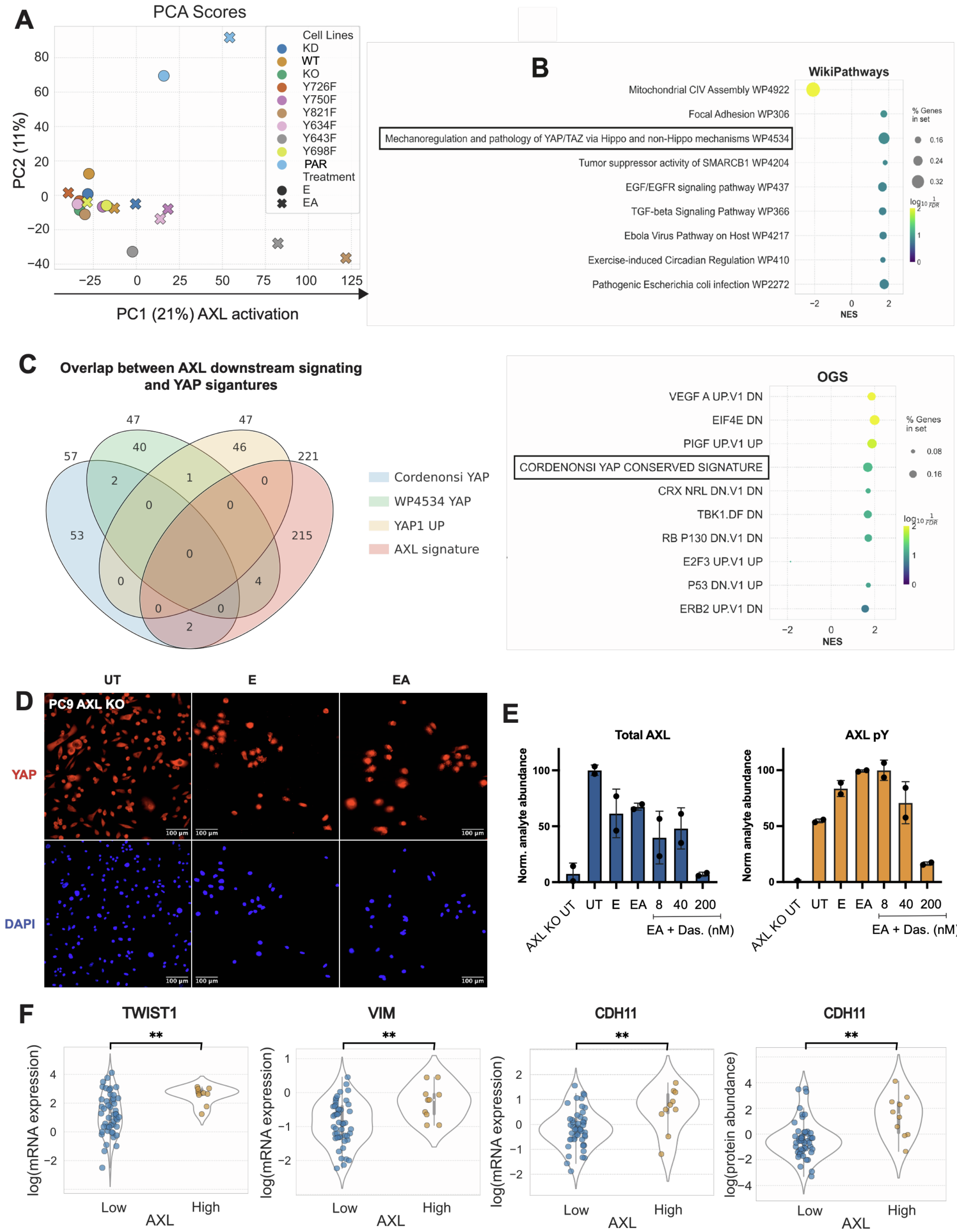

Figure S6. (A) Scores of a PCA analysis of the RNAseq data of the PC9 Y-to-F mutant cell lines treated with E or EA. (B) Ranked GSEA analysis by ascending PC 1 loadings using the gene

libraries WikiPathways and Oncogenic signature gene sets (OGS). (C) Venn diagram showing the overlap between the indicated AXL and YAP signatures. AXL signature refers to the genes present in C2 and C3 (**Fig. S3F**). (D) Immunofluorescence staining of YAP and DAPI in AXL KO cells. Scale bars represent 100 µm. (E) Luminex of AXL protein and phosphorylation levels in PC9 PAR and AXL KO cells exposed to the indicated treatments for 4h and 10 minutes of A. (F) Expression of mesenchymal markers by AXL levels. **p-value < 0.001 according to Student's t-test. Related to **Figure 6**.

REFERENCES

1. Majumder, A., Hosseinian, S., Stroud, M., Adhikari, E., Saller, J.J., Smith, M.A., Zhang, G., Agarwal, S., Creixell, M., Meyer, B.S., et al. (2022). Integrated Proteomics-Based Physical and Functional Mapping of AXL Kinase Signaling Pathways and Inhibitors Define Its Role in Cell Migration. Molecular Cancer Research, 1–14. https://doi.org/10.1158/1541-7786.mcr-21-0275.

2. Meyer, A.S., Zweemer, A.J.M., and Lauffenburger, D.A. (2015). The AXL Receptor Is a Sensor of Ligand Spatial Heterogeneity. Cell Systems *1*, 25–36. https://doi.org/10.1016/j.cels.2015.06.002.

3. Zweemer, A.J.M., French, C.B., Mesfin, J., Gordonov, S., Meyer, A.S., and Lauffenburger, D.A. (2017). Apoptotic Bodies Elicit Gas6-Mediated Migration of AXL-Expressing Tumor Cells. Molecular Cancer Research. https://doi.org/10.1158/1541-7786.mcr-17-0012.

4. Creixell, M., and Meyer, A.S. (2022). Dual data and motif clustering improves the modeling and interpretation of phosphoproteomic data. Cell Rep. methods *2*, 100167. https://doi.org/10.1016/j.crmeth.2022.100167.
